# Can people identify original and manipulated photos of real-world scenes?

**DOI:** 10.1186/s41235-017-0067-2

**Published:** 2017-07-18

**Authors:** Sophie J. Nightingale, Kimberley A. Wade, Derrick G. Watson

**Affiliations:** Department of Psychology, University of Warwick, Coventry, CV4 7AL UK

**Keywords:** Photo manipulation, Visual processing, Real-world scenes, Digital image forensics, Psychology and law

## Abstract

Advances in digital technology mean that the creation of visually compelling photographic fakes is growing at an incredible speed. The prevalence of manipulated photos in our everyday lives invites an important, yet largely unanswered, question: Can people detect photo forgeries? Previous research using simple computer-generated stimuli suggests people are poor at detecting geometrical inconsistencies within a scene. We do not know, however, whether such limitations also apply to real-world scenes that contain common properties that the human visual system is attuned to processing. In two experiments we asked people to detect and locate manipulations within images of real-world scenes. Subjects demonstrated a limited ability to detect original and manipulated images. Furthermore, across both experiments, even when subjects correctly detected manipulated images, they were often unable to locate the manipulation. People’s ability to detect manipulated images was positively correlated with the extent of disruption to the underlying structure of the pixels in the photo. We also explored whether manipulation type and individual differences were associated with people’s ability to identify manipulations. Taken together, our findings show, for the first time, that people have poor ability to identify whether a real-world image is original or has been manipulated. The results have implications for professionals working with digital images in legal, media, and other domains.

**Electronic supplementary material:**

The online version of this article (doi:10.1186/s41235-017-0067-2) contains supplementary material, which is available to authorized users.

## Significance

In the digital age, the availability of powerful, low-cost editing software means that the creation of visually compelling photographic fakes is growing at an incredible speed—we live in a world where nearly anyone can create and share a fake image. The rise of photo manipulation has consequences across almost all domains, from law enforcement and national security through to scientific publishing, politics, media, and advertising. Currently, however, scientists know very little about people’s ability to distinguish between original and fake images—the question of whether people can identify when images have been manipulated and what has been manipulated in the images of real-world scenes remains unanswered. The importance of this question becomes evident when considering that, more often than not, in today’s society we still rely on people to make judgments about image authenticity. This reliance applies to almost all digital images, from those that are used as evidence in the courtroom to those that we see every day in newspapers and magazines. Therefore, it is critical to better understand people’s ability to accurately identify fake from original images. This understanding will help to inform the development of effective guidelines and practices to address two key issues: how to better protect people from being fooled by fake images, and how to restore faith in original images.

## Background

In 2015, one of the world’s most prestigious photojournalism events—The World Press Photo Contest—was shrouded in controversy following the disqualification of 22 entrants, including an overall prize winner, for manipulating their photo entries. News of the disqualifications led to a heated public debate about the role of photo manipulation in photojournalism. World Press Photo responded by issuing a new code of ethics for the forthcoming contest that stipulated entrants “must ensure their pictures provide an accurate and fair representation of the scene they witnessed so the audience is not misled” (World Press Photo). They also introduced new safeguards for detecting manipulated images, including a computerized photo-verification test for entries reaching the penultimate round of the competition. The need for such a verification process highlights the difficulties competition organizers face in trying to authenticate images. If photography experts can’t spot manipulated images, what hope is there for amateur photographers or other consumers of photographic images? This is the question we aimed to answer. That is, to what extent can lay people distinguish authentic photos from fakes?

Digital image and manipulation technology has surged in the previous decades. People are taking more photos than ever before. Estimates suggested that one trillion photos would be taken in 2015 alone (Worthington, 2014), and that, on average, more than 350 million photos per day are uploaded to Facebook—that is over 14 million photos per hour or 4000 photos per second (Smith, 2013). Coinciding with this increased popularity of photos is the increasing frequency with which they are being manipulated. Although it is difficult to estimate the prevalence of photo manipulation, a recent global survey of photojournalists found that 76% regard photo manipulation as a serious problem, 51% claim to always or often enhance in-camera or RAW (i.e., unprocessed) files, and 25% admit that they, at least sometimes, alter the content of photos (Hadland, Campbell, & Lambert, 2015). Together these findings suggest that we are regularly exposed to a mix of real and fake images.

The prevalence and popularity of manipulated images raises two important questions. First, to what extent do manipulated images alter our thinking about the past? We know that images can have a powerful influence on our memories, beliefs, and behavior (e.g., Newman, Garry, Bernstein, Kantner, & Lindsay, 2012; Wade, Garry, Read, & Lindsay, 2002; Wade, Green, & Nash, 2010). Merely viewing a doctored photo and attempting to recall the event it depicts can lead people to remember wholly false experiences, such as taking a childhood hot-air balloon ride or meeting the Warner Brothers character Bugs Bunny at Disneyland (Braun, Ellis, & Loftus, 2002; Sacchi, Agnoli, & Loftus, 2007; Strange, Sutherland, & Garry, 2006). Thus, if people cannot differentiate between real and fake details in photos, manipulations could frequently alter what we believe and remember.

Second, to what extent should photos be admissible as evidence in court? Laws governing the use of photographic evidence in legal cases, such as the Federal Rules of Evidence (1975), have not kept up with digital change (Parry, 2009). Photos were once difficult to manipulate; the process was complex, laborious, and required expertise. Yet in the digital age, even amateurs can use sophisticated image-editing software to create detailed and compelling fake images. The Federal Rules of Evidence state that the content of a photo can be proven if a witness confirms it is fair and accurate. Put another way, the person who took the photo, any person who subsequently handles it, or any person present when the photo was taken, is not required to testify about the authenticity of the photo. If people cannot distinguish between original and fake photos, then litigants might use manipulated images to intentionally deceive the court, or even testify about images, unaware they have been changed.

Unfortunately, there is no simple solution to prevent people from being fooled by manipulated photos in everyday life or in the criminal arena (Parry, 2009). But the newly emerging field of image forensics is making it possible to better protect against photo fraud (e.g., Farid, 2006). Image forensics uses digital technology to determine image authenticity, and is based on the premise that digital manipulation alters the values of the pixels that make up an image. Put simply, the act of manipulating a photo leaves behind a trace, even if only subtle and not visible to the naked eye (Farid, 2009). Given that different types of manipulations—for instance, cloning, retouching, splicing—affect the underlying pixels in unique and systematic ways, image forensic experts can develop computer methods to reveal image forgeries. Such technological developments are being implemented in several domains, including law, photojournalism, and scientific publishing (Oosterhoff, 2015). The vast majority of image authenticity judgments, however, are still made by eye, and to our knowledge only one published study has explored the extent to which people can detect inconsistencies in images.

Farid and Bravo (2010) investigated how well people can make use of three cues— shadows, reflections, and perspective distortion—that are often indicative of photo tampering. The researchers created a series of computer-generated scenes consisting of basic geometrical shapes. Some scenes, for instance, were consistent with a single light source whereas others were inconsistent with a single light source. When the inconsistencies were obvious, that is, when shadows ran in opposite directions, observers were able to identify tampering with nearly 100% accuracy. Yet when the inconsistencies were subtle, for instance, where the shadows were a combination of results from two different light positions on the same side of the room, observers performed only slightly better than chance. These preliminary findings, based on computer-generated scenes of geometric objects, suggest that the human visual system is poor at identifying inconsistencies in such images.

In the current study we examined whether people are similarly poor at detecting inconsistencies within images of *real-world* scenes. On the one hand, we might expect people to perform even worse if trying to detect manipulations in real-world photos. Research shows that real-world photos typically contain many multi-element objects that can obscure distortions (Bex, 2010; Hulleman & Olivers, 2015). For example, people with the visual impairment metamorphopsia often do not notice any problems with their vision in their everyday experiences, yet the impairment is quite apparent when they view simple stimuli, such as a grid of evenly spaced horizontal and vertical lines (Amsler, 1953; Bouwens & Meurs, 2003). We also know that people find it more difficult to detect certain types of distortions, such as changes to image contrast, in complex real-world scenes than in more simplistic stimuli (Bex, 2010; Bex, Solomon, & Dakin, 2009). In sum, if people find it particularly difficult to detect manipulations in complex real-world scenes, then we might expect our subjects to perform worse than Farid and Bravo’s (2010) subjects.

On the other hand, there is good reason to predict that people might do well at detecting manipulations in real-world scenes. Visual cognition research suggests that people might detect image manipulations using their knowledge of the typical appearance of real-world scenes. Real-world scenes share common properties, such as the way the luminance values of the pixels are organized and structured (Barlow, 1961; Gardner-Medwin & Barlow, 2001; Olshausen & Field, 2000). Over time, the human visual system has become attuned to such statistical regularities and has expectations about how scenes *should* look. When an image is manipulated, the structure of the image properties change, which can create a mismatch between what people see and what they expect to see (Craik, 1943; Friston, 2005; Rao & Ballard, 1999; Tolman, 1948). Thus, based on this real-world scene statistics account, we might predict that people should be able to use this “mismatch” as a cue to detecting a manipulation. If so, our subjects should perform better than chance at detecting manipulations in real-world scenes.

Although there is a lack of research directly investigating the applied question of people’s ability to detect photo forgeries, people’s ability to detect change in a scene is well-studied in visual cognition. Notably, change blindness is the striking finding that, in some situations, people are surprisingly slow, or entirely unable, to detect changes made to, or find differences between, two scenes (e.g., Pashler, 1988; Simons, 1996; Simons & Levin, 1997). In some of the early studies, researchers demonstrated observers’ inability to detect changes made to a scene during an eye movement (saccade) using very simple stimuli (e.g., Wallach & Lewis, 1966), and later, in complex real-world scenes (e.g., Grimes, 1996). Researchers have also shown that change blindness occurs even when the eyes are fixated on the scene: The flicker paradigm, for instance, simulates the effects of a saccade or eye blink by inserting a blank screen between the continuous and sequential presentation of an original and changed image (Rensink, O’Regan, & Clark, 1997). It often requires a large number of alternations between the two images before the change can be identified. Furthermore, change blindness persists when the original and changed images are shown side by side (Scott-Brown, Baker, & Orbach, 2000), when change is masked by a camera cut in motion pictures (Levin & Simons, 1997), and even when change occurs in real-world situations (Simons & Levin, 1998).

Such striking failures of perception suggest that people do not automatically form a complete and detailed visual representation of a scene in memory. Therefore, to detect change, it might be necessary to draw effortful, focused attention to the changed aspect (Simons & Levin, 1998). So which aspects of a scene are most likely to gain focused attention? One suggestion is that attention is guided by salience; the more salient aspects of a scene attract attention and are represented more precisely than less salient aspects. In support of this idea, research has shown that changes to more important objects are more readily detected than changes made to less important objects (Rensink et al., 1997). Other findings, however, indicate that observers sometimes miss even large changes to central aspects of a scene (Simons & Levin, 1998). Therefore, the question of what determines scene saliency continues to be explored. Specifically, researchers disagree about whether the low-level visual salience of objects in a scene, such as brightness (e.g., Lansdale, Underwood, & Davies, 2010; Pringle, Irwin, Kramer, & Atchley, 2001; Spotorno & Faure, 2011) or the high-level semantic meaning of the scene (Stirk & Underwood, 2007) has the most influence on attentional allocation.

What other factors affect people’s susceptibility to change blindness? One robust finding in the signal detection literature is that the ability to make accurate perceptual decisions is related to the strength of the signal and the amount of noise (Green & Swets, 1966). Signal detection theory has been applied to change detection. In one study, observers judged whether two sequentially presented arrays of colored dots remained identical or if there was a change (Wilken & Ma, 2004). Crucially, the researchers manipulated the strength of the signal in the change trials by varying the number of colored dots in the display that changed, while noise (total set size) remained constant. Performance improved as a function of the number of dots in the display that changed color—put simply, greater signal resulted in greater change detection.

Given the lack of research investigating people’s ability to detect photo forgeries, change blindness offers a highly relevant area of research. A key difference between the change blindness research and our current experiments, however, is that our change detection task does not involve a comparison of two images; therefore, representing the scene in memory is not a factor in our research. That is, subjects do not compare the original and manipulated versions of an image. Instead, they make their judgment based on viewing only a single image. This image is either the original, unaltered image or an image that has been manipulated in some way.

In the current study, we explored people’s ability to identify common types of image manipulations that are frequently applied to real-world photos. We distinguished between physically implausible versus plausible manipulations. For example, a physically implausible image might depict an outdoor scene lit only by the sun with a person’s shadow running one way and a car’s shadow running the other way. Such shadows imply the impossible: two suns. Alternatively, when an unfamiliar face is retouched in an image it is quite plausible; eliminating spots and wrinkles or whitening teeth do not contradict physical constraints in the world that govern how faces ought to look. In our study, *geometrical* and *shadow* manipulations made up our implausible manipulation category, while *airbrushing* and *addition* or *subtraction* manipulations made up our plausible manipulation category. Our fifth manipulation type, *super-additive*, presented all four manipulation types in a single image and thus included both categories of manipulation.

We had a number of predictions about people’s ability to detect and locate manipulations in real-world photos. We expected the type of manipulation—implausible versus plausible—to affect people’s ability to detect and locate manipulations. In particular, people should correctly identify more of the physically implausible manipulations than the physically plausible manipulations given the availability of evidence within the photo. We also expected people to be better at correctly detecting and locating manipulations that caused more change to the pixels in the photo than manipulations that caused less change.

## Experiment 1

### Methods

#### Subjects and design

A total of 707 (*M* = 25.8 years, *SD* = 8.8, range = 14–82; 460 male, 226 female, 21 declined to respond) subjects completed the task online. A further 17 subjects were excluded from the analyses because they had missing response time data for at least one response on the detection or location task. There were no geographical restrictions and subjects did not receive payment for taking part, but they did receive feedback on their performance at the end of the task. Subject recruitment stopped when we reached at least 100 responses per photo. We used a within-subjects design in which each person viewed a series of ten photos, half of which had one of five manipulation types applied, and half of which were original, non-manipulated photos. We measured people’s accuracy in determining whether a photo had been manipulated or not and their ability to locate manipulations.

#### Stimuli

**Fig. 1:**
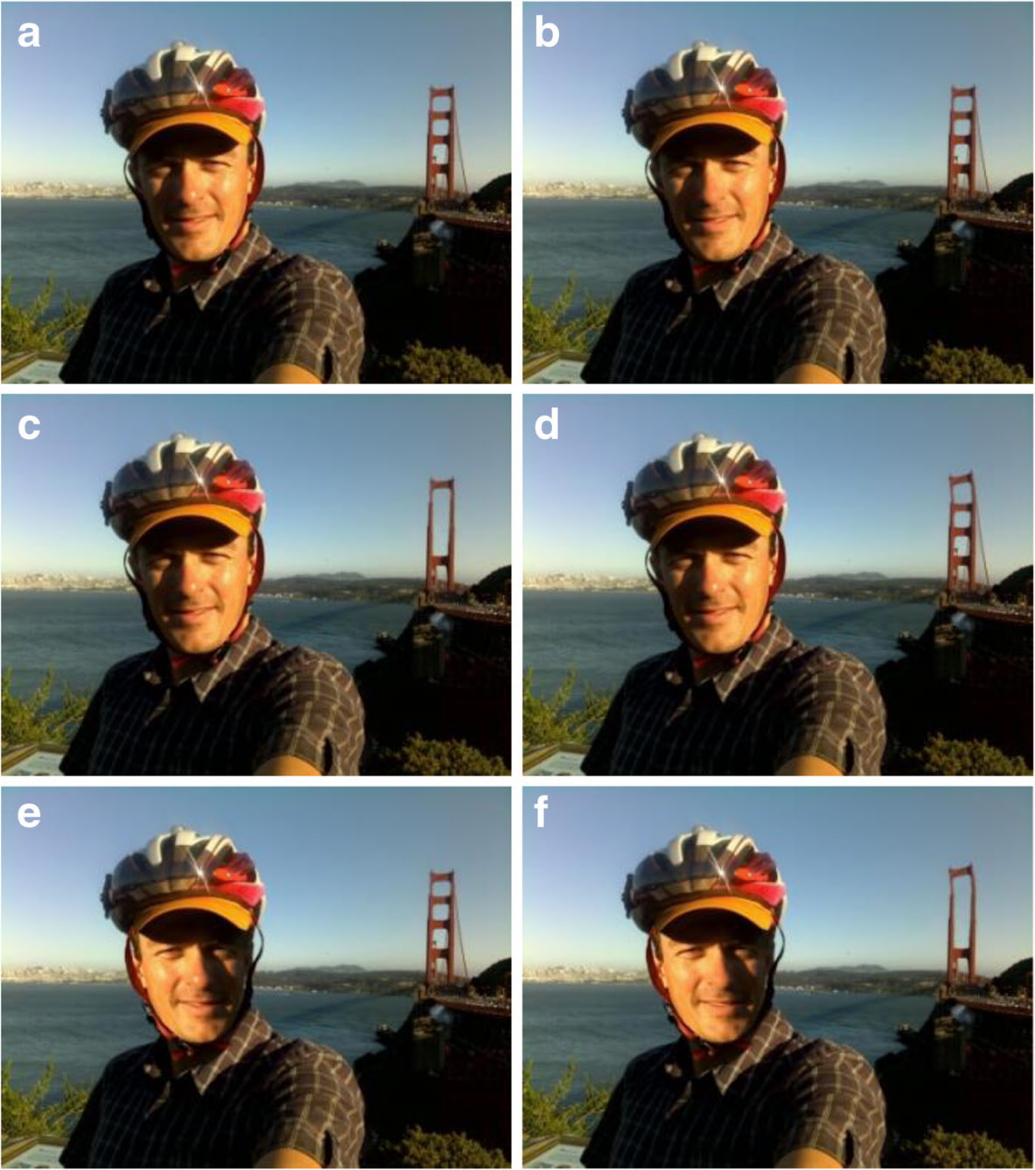
Samples of manipulated photos. **a** Original photo; **b** airbrushing—removal of sweat on the nose, cheeks, and chin, and removal of wrinkles around the eyes; **c** addition or subtraction—two links between the columns of the tower of the suspension bridge removed; **d** geometrical inconsistency—top of the bridge is sheered at an angle inconsistent with the rest of the bridge; **e** shadow inconsistency—face is flipped around the vertical axis so that the light is on the wrong side of the face compared with lighting in the rest of the scene; **f** super-additive—combination of all previously described manipulations. Original photo credit: Vin Cox, CC BY-SA 3.0 license. Photos **b**–**f** are derivatives of the original and licensed under CC BY-SA 4.0

We obtained ten colored images (JPEG format), 1600 × 1200 pixels, that depicted people in real-world scenes from Google Image search (permitted for non-commercial re-use with modification). The first author (SN) used GNU Image Manipulation Program (GIMP) to apply five different, commonly used manipulation techniques: (a) airbrushing, (b) addition or subtraction, (c) geometrical inconsistency, (d) shadow inconsistency, and (e) super-additive (manipulations a to d included within a single image). For the airbrushing technique, we changed the person’s appearance by whitening their teeth, removing spots, wrinkles, or sweat, or brightening their eye color. For the addition or subtraction technique, we added or removed objects, or parts of objects. For example, we removed links between tower columns on a suspension bridge and inserted a boat into a river scene. For geometrical inconsistencies, we created physically implausible changes, such as distorting angles of buildings or sheering trees in different directions to others to indicate inconsistent wind direction. For shadow inconsistencies, we removed or changed the direction of a shadow to make it incompatible with the remaining shadows in the scene. For instance, flipping a person’s face around the vertical axis causes the shadow to appear on the wrong side compared with the rest of the body and scene. In the super-additive technique we presented all four previously described manipulation types in one photo. Figure 1 shows examples of the five manipulation types, and higher resolution versions of these images, as well as other stimuli examples, appear in Additional file 1.

In total, we had ten photos of different real-world scenes. The non-manipulated version of each of these ten photos was used to create our original photo set. To generate the manipulated photos, we applied each of the five manipulation types to six of the ten photos, creating six versions of each manipulation for a total of 30 manipulated photos. This gave us an overall set of 40 photos. Subjects saw each of the five manipulation types and five original images but always on a different photo.

Image-based saliency cues can determine where subjects direct their attention; thus, we checked whether our manipulations had changed the salience of the manipulated area within the image. To examine this, we ran the images through two independent saliency models: the classic Itti-Koch model (Itti & Koch, 2000; Itti, Koch, & Niebur, 1998) and the Graph-Based Visual Saliency (GBVS) model (Harel, Koch, & Perona, 2006). To summarize, we found that our manipulations did not inadvertently change the salience of the manipulated regions. See Additional file 2 for details of these analyses.

#### Procedure

**Fig. 2:**
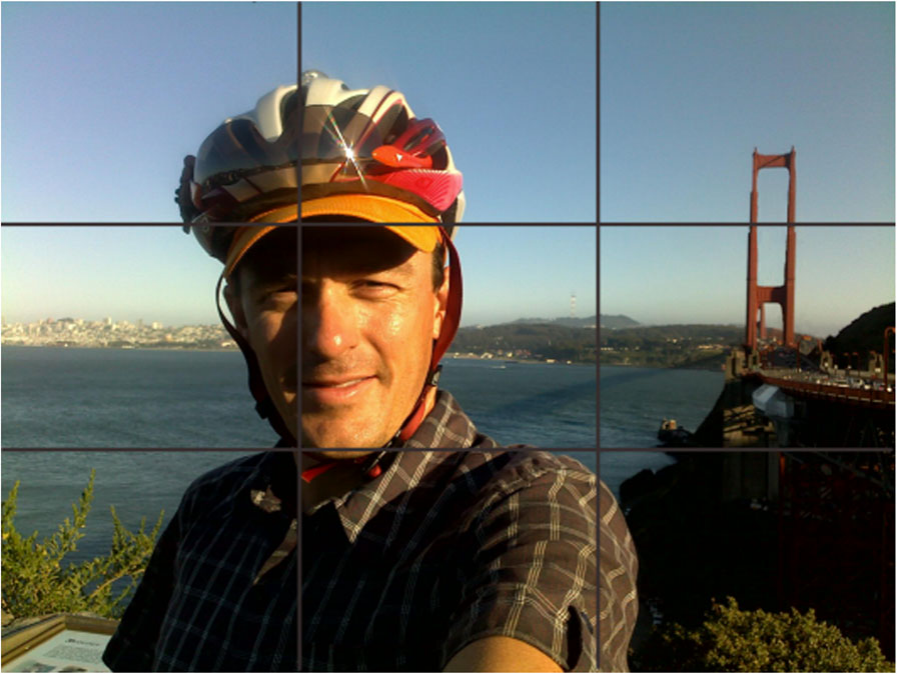
Example of a photo with the location grid overlaid. Photo credit: Vin Cox, CC BY-SA 3.0 license

Subjects answered questions about their demographics, attitudes towards image manipulation, and experiences of taking and manipulating photos. Subjects were then shown a practice photo and instructed to adjust their browser zoom level so that the full image was visible. Next, subjects were presented with ten photos in a random order and they had an unlimited amount of time to view and respond to each photo. We first measured subjects’ ability to *detect* whether each photo had been manipulated by asking “Do you think this photograph has been digitally altered?” Subjects were given three response options: (a) “Yes, and I can see exactly where the digital alteration has been made”; (b) “Yes, but I cannot see specifically what has been digitally altered”; or (c) “No.” For the manipulated photos, we considered either of the “yes” responses as correct; for original photos we considered “no” as correct. Following a “yes” response, we immediately measured subjects’ ability to *locate* the manipulation by presenting the same photo again with a 3 × 3 grid overlaid^1^ (see Fig. 2 for an example). Subjects were asked to: “Please select the box that you believe contains the digitally altered area of the photograph (if you believe that more than one region contains digital alteration, please select the one you feel contains the majority of the change).” On average, manipulations spanned two regions in the grid. For the analyses we considered a response to be correct if the subject clicked on a region that contained any of the manipulated area or a nearby area that could be used as evidence that a manipulation had taken place—a relatively liberal criterion. Subjects received feedback on their performance at the end of the study.

### Results and discussion

An analysis of the response time data suggested that subjects were engaged with the task and spent a reasonable amount of time determining which photos were authentic. In the detection task, the mean response time per photo was 43.8 s (*SD* = 73.3 s) and the median response time 30.4 s (interquartile range 21.4, 47.7 s). In the location task, the mean response time was 10.5 s (*SD* = 5.7 s) and the median response time 9.1 s (interquartile range 6.5, 13.1 s). Following Cumming’s (2012) recommendations, we present our findings in line with the estimation approach by calculating a precise estimate of the actual size of the effects.

#### Overall accuracy on the detection task and the location task

We now turn to our primary research question: To what extent can people detect and locate manipulations of real-world photos? For the detection task, we collapsed across the two “yes” response options such that if subjects responded either “Yes, and I can see exactly where the digital alteration has been made” or “Yes, but I cannot see specifically what has been digitally altered”, then we considered this to be a “yes” response. Thus, chance performance was 50%. Overall performance on the detection task was better than chance; a mean 66% of the photos were correctly classified as original or manipulated, 95% confidence interval (CI)^2^ [65%, 67%]. Subjects’ ability to distinguish between original (72% correct) and manipulated (60% correct) photos of real-world scenes was reliably greater than zero, discrimination (*d'*) = 0.80, 95% CI [0.74, 0.85]. Moreover, subjects showed a bias towards saying that photos were real; response bias (*c*) = 0.16, 95% CI [0.12, 0.19]. Although subjects’ ability to detect manipulated images was above chance, it was still far from perfect. Furthermore, even when subjects correctly indicated that a photo had been manipulated, they could not necessarily locate the manipulation. Collapsing over all manipulation types, a mean 45% of the manipulations were accurately located, 95% CI [43%, 46%]. To determine chance performance in the location task, we need to take into account that subjects were asked to select one of nine regions of the image. Therefore, subjects had less chance of being correct by guessing in the location task than the detection task. On average, the manipulations were contained within two of the nine regions. But because the chance of being correct by guessing varied for each image and each manipulation type, we ran a Monte Carlo simulation to determine the chance rate of selecting the correct region. Table 1 shows the results from one million simulated responses. Overall, chance performance was 24%; therefore, collectively, subjects performed better than chance on the location task. Overall, the results show that people have some (above chance) ability to detect and locate manipulations, although performance is far from perfect.

**Table 1:** Mean number of regions (out of a possible nine) containing manipulation and results of Monte Carlo simulation to determine chance performance in location task by manipulation type and overall

Manipulation type	Number of regions	Percentage correct by chance	
	*M*	*M*	95% CI
Airbrushing	1.83	20	[20, 21]
Add/sub	1.33	17	[17, 17]
Geometry	1.5	19	[18, 19]
Shadow	1.67	15	[15, 15]
Super-additive	4.33	48	[48, 48]
Overall	2.13	24	[24, 24]

*CI* confidence interval. For each manipulation type, we show the mean number of regions that contained the manipulation across all six images. The manipulation type “Overall” is the mean number of manipulated regions across all six images and all five manipulation types. To determine chance performance in the location task, we ran a Monte Carlo simulation of one million responses based on the number of regions manipulated for each image and manipulation type

#### Ability to detect and locate by manipulation type

**Fig. 3:**
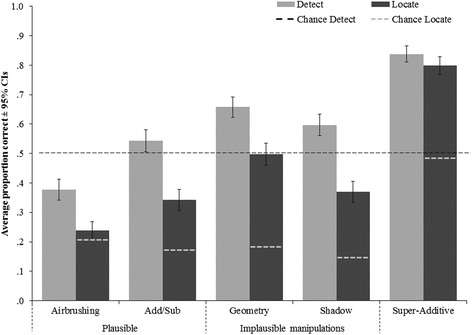
Mean proportion of correct “detect” and “locate” decisions by type of photo manipulation. The *dotted line* represents chance performance for detection. The *grey dotted lines* on the *locate bars* represent chance performance by manipulation type in the location task. *Error bars* represent 95% CIs

We predicted that people’s ability to detect and locate manipulations might vary according to the manipulation type. Figure 3 shows subjects’ accuracy on both the detection and the location task by manipulation type. In line with our prediction, subjects were better at detecting manipulations that included physically implausible changes (geometrical inconsistencies, shadow inconsistencies, and super-additive manipulations) than images that included physically plausible changes (airbrushing alterations and addition or subtraction of objects).

**Fig. 4:**
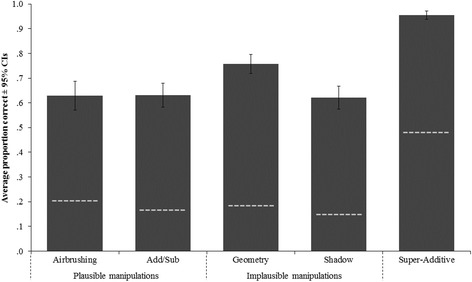
Mean proportion of correct “locate” decisions when subjects correctly detected that the photo was manipulated (i.e., correctly said “Yes” on the detection task). The *grey dotted lines* on the *bars* represent chance performance for each manipulation type. *Error bars* represent 95% CIs

It was not the case, however, that subjects were necessarily better at locating the manipulation within the photo when the change was physically implausible. Figure 4 shows the proportion of manipulated photo trials in which subjects correctly detected a manipulation and also went on to correctly locate that manipulation, by manipulation type. Across both physically implausible and physically plausible manipulation types, subjects often correctly indicated that photos were manipulated but failed to then accurately locate the manipulation. Furthermore, although the physically implausible geometrical inconsistencies were more often correctly located, the shadow inconsistencies were only located equally as often as the physically plausible manipulation types—airbrushing and addition or subtraction. These findings suggest that people may find it easier to detect physically implausible, rather than plausible, manipulations, but this is not the case when it comes to locating the manipulation.

#### Image metrics and accuracy

To understand more about people’s ability to identify image manipulations, we examined how the amount of change in a photo affects people’s accuracy in the detection and location tasks. When an image is digitally altered, the structure of the underlying elements—the pixels—are changed. This change can be quantified in numerous ways but we chose to use Delta-E_76_ because it is a measure based on both color and luminance (Robertson, 1977). To calculate Delta-E, we first converted the images in Matlab® to L*a*b* color space because it has a dimension for lightness as well as color. Next we calculated the difference between corresponding pixels in the original and manipulated versions of each photo. Finally, these differences were averaged to give a single Delta-E score for each manipulated photo. A higher Delta-E value indicates a greater amount of difference between the original and the manipulated photo.^3^ We calculated Delta-E for each of the 30 manipulated photos.

**Fig. 5:**
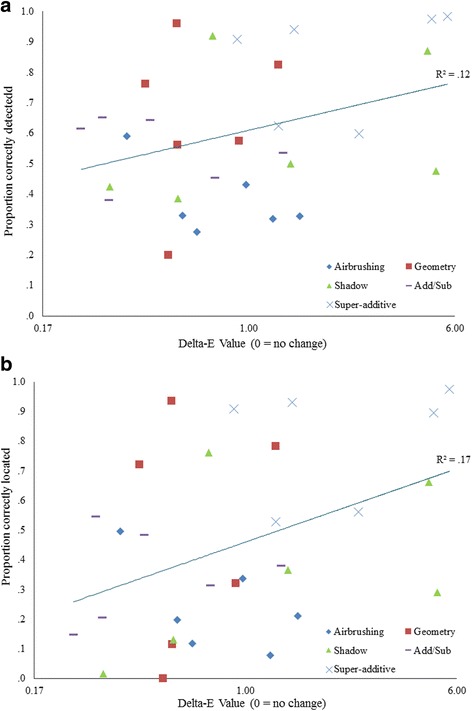
Mean proportion of correctly detected (**a**) and located (**b**) image manipulations by extent of pixel distortion as measured by Delta-E. The graphs show individual data points for each of the 30 manipulated images

Figure 5 shows the log Delta-E values on the x-axis, where larger values indicate more change in the color and luminance values of pixels in the manipulated photos compared with their original counterpart. The proportions of correct detection (Fig. 5a) and location (Fig. 5b) responses for each of the manipulated photos are presented on the y-axis. We found a positive relationship between the Delta-E measure and the proportion of photos that subjects correctly detected as manipulated, albeit not reaching significance: *r*(28) = 0.34, *p* = 0.07.^4^ Furthermore, the Delta-E measure was positively correlated with the proportion of manipulations that were correctly located, *r*(28) = 0.41, *p* = 0.03. As predicted, these data suggest that people might be sensitive to the low level properties of real-world scenes when making judgments about the authenticity of photos. This finding is especially remarkable given that our subjects never saw the same scene more than once and so never saw the original version of a manipulated image. This finding fits with the proposition that disrupting the underlying pixel structure might exacerbate the difference between the manipulated photos and people’s expectations of how a scene should look. Presumably, these disruptions make it easier for people to accurately classify manipulated photos as being manipulated. We can also interpret these findings based on a signal detection account—adding greater signal (in our experiment, more change to an image, as measured by Delta-E) results in greater detection of that signal (Green & Swets, 1966; Wilken & Ma, 2004).

**Fig. 6:**
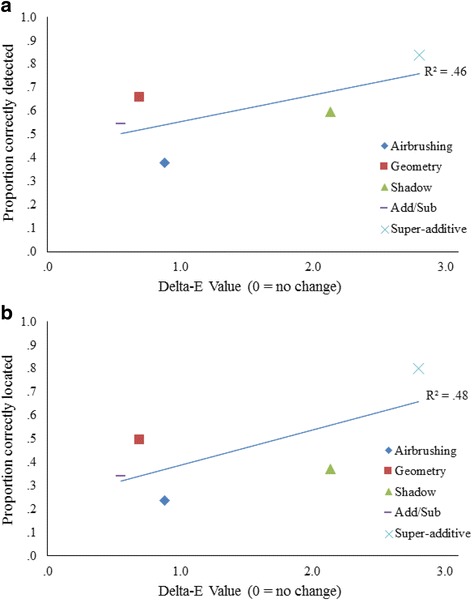
Mean proportion of correctly detected (**a**) and located (**b**) image manipulations by extent of pixel distortion as measured by Delta-E. The graphs show the mean values for each of the five categories of manipulation type

Next, we tested whether there was a relationship between the mean amount of change and the mean proportion of correct detection (Fig. 6a) and location (Fig. 6b) responses by the category of manipulation type. As Fig. 6 shows, there was a numerical, but non-significant, trend for a positive relationship between amount of change and the proportion of photos that subjects correctly detected as manipulated: *r*(3) = 0.68, *p* = 0.21. There was also a numerical trend for a positive relationship between amount of change and the proportion of manipulations that were correctly located: *r*(3) = 0.69, *p* = 0.19.

#### Individual factors in detecting and locating manipulations

To determine whether individual factors play a role in detecting and locating manipulations, we gathered subjects’ demographic data, attitudes towards image manipulation, and experiences of taking and manipulating photos. We also recorded subjects’ response times on the detection and location tasks.

To determine how each factor influenced subjects’ performance on the manipulated image trials, we conducted two generalized estimating equation (GEE) analyses—one for accuracy on the detection task and one for accuracy on the location task. Specifically, we conducted a repeated measures logistic regression with GEE because our dependent variables were binary with both random and fixed effects (Liang & Zeger, 1986). For the detection task, we ran two additional repeated measures linear regression GEE models to explore the effect of the predictor variables on signal detection estimates *d'* and *c*. The results of the GEE analyses are shown in Table 2. In the detection task, faster responses were more likely to be associated with accurate responses than slower responses. There was also a small effect of people’s general belief about the prevalence of manipulated photos in their everyday lives on accuracy in the detection task. Those who believe a greater percentage of photos are digitally manipulated were more likely to correctly identify manipulated photos than those who believe a lower percentage of photos are digitally manipulated. Further, the results of the signal detection analysis suggest that this results from a difference in ability to discriminate between original and manipulated photos, rather than a shift in response bias—those who believe a greater percentage of photos are digitally manipulated accurately identified more of the manipulated photos without an increased false alarm rate. General beliefs about the prevalence of photo manipulation did not have an effect on people’s ability to locate the manipulation. This pattern of results is somewhat surprising. It seems intuitive to think that a general belief that manipulated photos are prevalent simply makes people more likely to report that a photo is manipulated because they are generally skeptical about the veracity of photos rather than because they are better at spotting fakes. Although interesting, the small effect size and counterintuitive nature of the finding indicate that it is important to replicate the result prior to drawing any strong conclusions. The only variable that had an effect on accuracy in the location task was gender; males were slightly more likely than females to correctly locate the manipulation within the photo.

**Table 2:** Results of the GEE binary logistic and linear regression models to determine variables that predict accuracy on the detect and locate tasks

Predictor	Detect			Locate		
	*B*	*OR 95% CI*	*p*	*B*	*OR 95% CI*	*p*
Response time						
Accuracy	0.11	1.11 [1.08, 1.15]	<0.001	-	-	-
*d'*	−0.01	0.99 [0.98, 1.01]	0.31	-	-	-
*c*	0.01	1.01 [1.00, 1.02]	0.10	-	-	-
General beliefs about percentage of images manipulated = High (71–100%)						
Accuracy	0.20	1.22 [1.06, 1.41]	0.01	0.11	1.11 [0.98, 1.26]	0.10
*d'*	0.16	1.17 [1.05, 1.30]	0.01	-	-	-
*c*	−0.05	0.96 [0.90, 1.02]	0.16	-	-	-
Gender = Female						
Accuracy	0.05	1.05 [0.90, 1.23]	0.50	−0.16	0.86 [0.75, 0.98]	0.03
*d'*	−0.06	0.95 [0.84, 1.06]	0.35	-	-	-
*c*	−0.05	0.95 [0.89, 1.02]	0.15	-	-	-
Interest in photography = Interested						
Accuracy	0.06	1.07 [0.92, 1.24]	0.41	0.04	1.05 [0.92, 1.19]	0.51
*d'*	−0.02	0.98 [0.88, 1.10]	0.73	-	-	-
*c*	−0.05	0.96 [0.89, 1.03]	0.20	-	-	-
Frequency of taking photos = Daily/weekly						
Accuracy	−0.15	0.86 [0.73, 1.01]	0.07	−0.07	0.94 [0.81, 1.08]	0.35
*d'*	−0.08	0.92 [0.81, 1.04]	0.18	-	-	-
*c*	0.01	1.01 [0.94, 1.09]	0.71	-	-	-

B and odds ratios (OR) estimate the degree of change in (a) accuracy on the task (based on the manipulated image trials), (b) *d'*, or (c) *c* associated with one unit change in the independent variable. An odds ratio of 1 indicates no effect of the independent variable on accuracy; values of 1.5, 2.5, and 4.0 are generally considered to reflect small, medium, and large effect sizes, respectively (Rosenthal, 1996). The category order for factors was set to descending to make the reference level 0. The reference groups are: General beliefs about percentage of images manipulated = Low (0–70%), Gender = Male, Interest in photography = Not Interested, Frequency of taking photos = Monthly/yearly/never. For response time (RT) we divided the data into eight equal groups (level 1 represents the slowest RTs (≥43.4 s) and level 8 the fastest RTs (≤8.4 s)). The 21 subjects who chose not to disclose their gender were excluded from these analyses leaving a total sample of *n* = 686. Given that subjects only responded on the location task if they said “yes”, the photo had been manipulated, we did not have location response time data for all of the trials and therefore were unable to consider response time on the location task. Because we did not have a fixed number of choices per condition in the location task, we were unable to calculate the degree of change in *d'* or *c* associated with the predictor variables

Together these findings show that individual factors have relatively little impact on the ability to detect and locate manipulations. Although shorter response times were associated with more correct detections of manipulated photos, we did not manipulate response time so we cannot know whether response time affects people’s ability to discriminate between original and manipulated photos. In fact, our response time findings might be explained by a number of perceptual decision making models, for example, the drift diffusion model (Ratcliff, 1978). However, determining the precise mechanism that accounts for the association between shorter response times and greater accuracy is beyond the scope of the current paper.

Experiment 1 indicates that people have some ability to distinguish between original and manipulated real-world photos. People’s ability to correctly identify manipulated photos was better than chance, although not by much. Our data also suggest that locating photo manipulations is a difficult task, even when people correctly indicate that a photo is manipulated. We should note, however, that our study could have underestimated people’s ability to locate manipulations in real-world photos. Recall that subjects were only asked to locate manipulations on photos that they thought were manipulated. It remains possible people might be able to locate manipulations even if they do not initially think that a photo has been manipulated. We were unable to check this possibility in Experiment 1, so we addressed this issue in Experiment 2 by asking subjects to complete the location task for all photos, regardless of their initial response in the detection task. If subjects did not think that the photo had been manipulated, we asked them to make a guess about which area of the image might have been changed.

We also created a new set of photographic stimuli for Experiment 2. Rather than sourcing photos online, the first author captured a unique set of photos on a Nikon D40 camera in RAW format, and prior to any digital editing, converted the files to PNGs. There are two crucial benefits to using original photos rather than downloading photos from the web. First, by using original photos we could be certain that our images had not been previously manipulated in any way. Second, when digital images are saved, the data are compressed to reduce the file size. JPEG compression is lossy in that some information is discarded to reduce file size. This information is not generally noticeable to the human eye (except at very high compression rates when compression artifacts can occur); however, the process of converting RAW files to PNGs (a lossless format) prevented *any* loss of data in either the original or manipulated images and, again, ensured that our photos were not manipulated in any way before we intentionally manipulated them.

## Experiment 2

### Methods

#### Subjects and design

A total of 659 (*M* = 25.5 years, *SD* = 8.2, range = 13–70; 362 male, 283 female, 14 declined to respond) subjects completed the study online. A further 32 subjects were excluded from the analyses because they had missing response time data for at least one response on the detection or location task. As in Experiment 1, subjects did not receive payment for taking part but were given feedback on their performance at the end of the study. We stopped collecting data once we reached 100 responses per photo. The design was similar to that of Experiment 1.

#### Stimuli

We took our own photos in RAW format at a resolution of 3008 × 2000 pixels and converted them to PNGs with a resolution of 1600 × 1064 pixels prior to any digital editing. We checked the photos to ensure there were no spatial distortions caused by the lens, such as barrel or pincushion distortion. The photo manipulation process was the same as in Experiment 1. We applied the five manipulation techniques to six different photos to create a total of 30 manipulated photos. We used the non-manipulated version of these six photos and another four non-manipulated photos to give a total of ten original photos. Thus, the total number of photos was 40. As in Experiment 1, we ran two independent saliency models to check whether our manipulations had influenced the salience of the region where the manipulation had been made. See Additional file 2 for details of the saliency analyses. Similar to Experiment 1, our manipulations made little difference to the salience of the regions of the image.

#### Procedure

The procedure was similar to that used in Experiment 1, except for the following two changes. First, subjects were asked to locate the manipulation regardless of their response in the detection task. Second, subjects were asked to click on one of 12, rather than nine, regions on the photo to locate the manipulation. We increased the number of regions on the grid to ensure that the manipulations in the photos spanned two regions, on average, as per Experiment 1.

### Results and discussion

As in Experiment 1, subjects spent a reasonable amount of time examining the photos. In the detection task, the mean response time per photo was 57.8 s (*SD* = 271.5 s) and the median 24.3 s (interquartile range = 17.3 to 37.4 s). In the location task, the mean response time was 10.9 s (*SD* = 27.0 s) and the median 8.2 s (interquartile range = 6.1 to 11.2 s).

#### Overall accuracy on the detection task and the location task

Overall accuracy in the detection task was slightly lower than that observed in Experiment 1, but still above chance: Subjects correctly classified 62% of the photos as being original or manipulated (cf. 66% in Experiment 1), 95% CI [60%, 63%]. Subjects had some ability to discriminate between original (58% correct) and manipulated (65% correct) photos, *d'* = 0.56, 95% CI [0.50, 0.62], replicating the results from Experiment 1. Again, this provides some support for the prediction that the match or mismatch between the information in the photo and people’s expectation of what real-world scenes look like might help people to identify original and manipulated real-world photos. In contrast to Experiment 1, however, subjects did not show a bias towards saying that photos were authentic: *c* = −0.07, 95% CI [−0.10, −0.04]. It is possible that asking all subjects to search for evidence of a manipulation—the location task—regardless of their answer in the detection task, prompted a more careful consideration of the scene. In line with this account, subjects in Experiment 2 spent a mean of 14 s longer per photo on the detection task than those in Experiment 1.

Recall that the results from Experiment 1 suggested that subjects found the location task difficult, even when they correctly detected the photo as manipulated. Yet, we were unable to conclusively say that location was more difficult than detection because we did not have location data for the manipulated photo trials that subjects failed to detect. In Experiment 2 we gathered those data, but before we could directly compare subjects’ ability to detect manipulated photos with their ability to locate the manipulations within, we had to correct for guessing. For the detection task, chance performance was the same as Experiment 1, 50%. For the location task, however, there were two differences to Experiment 1. First, subjects were asked to select one of 12, rather than one of nine, image regions. Second, we used a new image set; thus, the number of regions manipulated for each image and manipulation type changed. Accordingly, we ran a separate Monte Carlo simulation to determine the chance rate of selecting the correct region. Table 3 shows that overall chance performance in the location task was 17%.

**Table 3:** Mean number of regions (out of a possible 12) containing manipulation and results of Monte Carlo simulation to determine chance performance in location task by manipulation type and overall

Manipulation type	Number of regions	Percentage correct by chance	
	*M*	*M*	95% CI
Airbrushing	1.50	12	[12, 13]
Add/sub	1.33	11	[11, 11]
Geometry	1.33	11	[11, 11]
Shadow	1.33	11	[11, 11]
Super-additive	4.67	39	[39, 39]
Overall	2.03	17	[17, 17]

*CI* confidence interval. For each manipulation type, we show the mean number of regions that contained the manipulation across all six images. The manipulation type “Overall” is the mean number of manipulated regions across all six images and all five manipulation types. To determine chance performance in the location task, we ran a Monte Carlo simulation of one million responses based on the number of regions manipulated for each image and manipulation type

Subjects performed better than chance on the location task: a mean 56% of the manipulations were accurately located, 95% CI [55%, 58%]. Given that a mean 62% of the manipulated images were accurately detected and a mean 56% of the manipulations located, it seems that performance was very roughly similar on the two tasks. But this interpretation doesn’t take into account how subjects would perform by chance alone. A fairer approach is to compare subjects’ performance on the detection and location tasks with chance performance on those two tasks. For the detection task, subjects detected a mean 12% more manipulated images than would be expected by chance alone, 95% CI [10%, 13%]. Yet, somewhat surprisingly, subjects located a mean 39% more of the manipulations than would be expected by chance alone, 95% CI [38%, 41%]. This finding suggests that people are better at the more direct task of locating manipulations than the more generic one of detecting if a photo has been manipulated or not. Although this potential distinction between people’s ability to detect and locate manipulations is an interesting finding, the reason for it is not immediately apparent. One possibility is that our assumption that each of the 12 image regions has an equal chance of being picked is too simplistic—perhaps certain image regions never get picked (e.g., a relatively featureless area of the sky). If so, including these never picked regions in our chance calculation might make subjects’ performance on the location task seem artificially high. To check this possibility, we ran a second chance performance calculation.

In Experiment 2, even when subjects did not think that the image had been manipulated, they still attempted to guess the region that had been changed. Therefore, we can use these localization decisions in the original (non-manipulated) versions of the six critical photos to determine chance performance in the task. This analysis allows us to calculate chance based on the regions (of non-manipulated images) that people actually selected when guessing rather than assuming each of the 12 regions has an equal chance of being picked. Using this approach, Table 4 shows that overall chance performance in the location task was 23%. Therefore, even based on this chance localization level, subjects still located a mean 33% more of the locations than would be expected by chance alone, 95% CI [32%, 35%]. This finding supports the idea that subjects are better at the more direct task of locating manipulations than detecting whether a photo has been manipulated or not.

**Table 4:** Chance performance in location task by manipulation type and overall based on mean number of subjects choosing the manipulated region in the original version of the image

Manipulation type	Percentage correct by chance						
	Image						
	A	B	C	D	E	F	Overall
Airbrushing	19	31	28	28	23	20	25
Add/sub	24	5	15	3	3	1	9
Geometry	11	12	17	2	26	12	13
Shadow	20	16	28	39	4	5	19
Super-additive	74	63	44	72	33	26	53
Overall image	30	25	27	29	18	13	23

For each of the six critical images and each of the five manipulation types, we show the probability that the manipulated region of the image was selected by chance in the original version of the image. The “Overall” column denotes the mean probability of selecting the manipulated regions for that manipulation type across all 6 images A-F. The “Overall image” is the mean probability of selecting the manipulated regions for that image across all manipulation types. Each image had a minimum of 101 responses

#### Ability to detect and locate manipulations

On the manipulated photo trials, asking subjects to locate the manipulation regardless of whether they correctly detected it allowed us to segment accuracy in the following ways: (i) accurately detected and accurately located (hereafter, *DL*), (ii) accurately detected but not accurately located (*DnL*), (iii) inaccurately detected but accurately located (*nDL*), or (iv) inaccurately detected and inaccurately located (*nDnL*). Intuitively, it seems most practical to consider the more conservative accuracy—DL—as correct, especially in certain contexts, such as the legal domain, where it is crucial to know not only that an image has been manipulated, but precisely what about it is fake. That said, it might be possible to learn from the DnL and nDL cases to try to better understand how people process manipulated images.

**Fig. 7:**
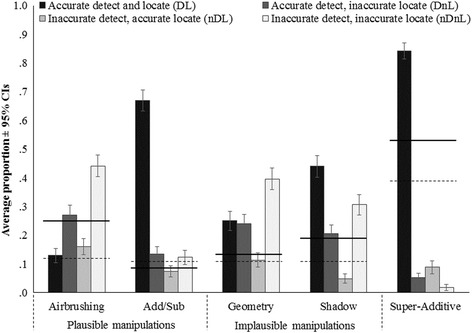
Mean proportion of manipulated photos accurately detected and accurately located (DL), accurately detected, inaccurately located (DnL), inaccurately detected, accurately located (nDL), and inaccurately detected, inaccurately located (nDnL) by manipulation type. The *dotted horizontal lines* on the *bars* represent chance performance for each manipulation type from the results of the Monte Carlo simulation. The full *horizontal lines* on the *bars* represent chance performance for each manipulation type based on subjects’ responses on the original image trials. *Error bars* represent 95% CIs

Figure 7 shows the proportion of DL, DnL, nDL, and nDnL responses for each of the manipulation types. The most common outcomes were for subjects to both accurately detect and accurately locate manipulations, or both inaccurately detect and inaccurately locate manipulations. It is interesting, however, that on almost a fifth (18%) of the manipulated photo trials, subjects accurately detected the photo as manipulated yet failed to locate the alteration. For 10% of the manipulated trials, subjects failed to detect but went on to successfully locate the manipulation. Subjects infrequently managed to detect and locate airbrushing manipulations; in fact it was more likely that subjects made DnL or nDL responses. Although this fits with our prediction that plausible manipulations would be more difficult to identify than implausible ones, the pattern of results for geometrical inconsistency, shadow inconsistency, and addition or subtraction do not support our prediction. Subjects made more DL responses on the plausible addition or subtraction manipulation photos than on either of the implausible types, geometrical manipulations and shadow manipulations. Why, then, are subjects performing better than expected by either of the chance measures on the addition or subtraction manipulations and worse than expected on the airbrushing ones? One possibility is that people’s ability to detect image manipulations is less to do with the plausibility of the change and more to do with the amount of physical change caused by the manipulation. We now look at this hypothesis in more detail by exploring the relationship between the image metrics and people’s ability to identify manipulated photos.

#### Image metrics and accuracy

**Fig. 8:**
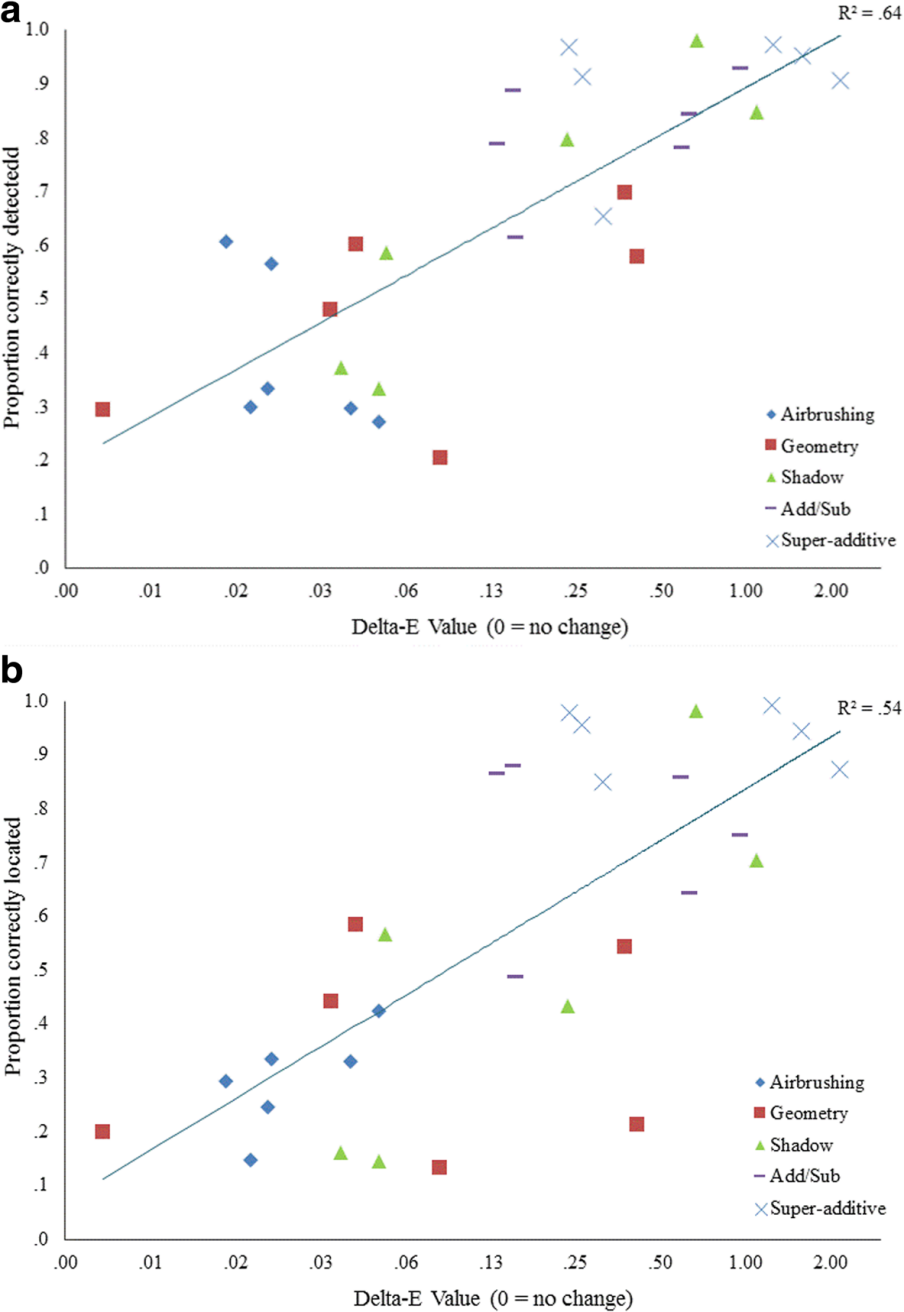
Mean proportion of correctly detected (**a**) and located (**b**) image manipulations by extent of pixel distortion as measured by Delta-E. The graphs show individual data points for each of the 30 manipulated images

Recall that the results from Experiment 1 suggested a relationship between the correct detection and location of image manipulations and the amount of disruption the manipulations had caused to the underlying structure of the pixels. Yet, the JPEG format of the images used in Experiment 1 created some (re-compression) noise in the Delta-E measurements between different images; thus, we wanted to test whether the same finding held with the lossless image format used in Experiment 2. As shown in Fig. 8, we found that the Delta-E measure was positively correlated with the proportion of photos that subjects correctly detected as manipulated (*r*(28) = 0.80, *p* < 0.001) and the proportion of manipulations that were correctly located (*r*(28) = 0.73, *p* < 0.001). These Pearson correlation coefficients are larger than those in Experiment 1 (cf. detect *r* = 0.34 and locate *r* = 0.41 in Experiment 1). It is possible that the re-compression noise in the JPEG images in Experiment 1 obscured the relationship between Delta-E and detection and localization performance. To check whether there was a stronger relationship between Delta-E and people’s ability to detect and locate image manipulations in Experiment 2 than Experiment 1, we converted the correlation coefficients to z values using Fisher’s transformation. There was a significantly stronger correlation between the Delta-E and detection in Experiment 2 than in Experiment 1: *z* = −2.74, *p* = 0.01. Yet because we had good reason to predict a stronger relationship in Experiment 2 than Experiment 1 (based on the JPEG re-compression noise), it might be fairer to consider the *p* value associated with a one-tailed test, *p* = 0.003. The correlation between Delta-E and accurate localization was not significantly stronger in Experiment 2 than in Experiment 1 based on a two-tailed test (*z* = −1.81, *p* = 0.07), but was based on a one-tailed test (*p* = 0.04). Therefore, it is possible that the global (re-compression) noise in the Delta-E values in Experiment 1 weakened the association between the amount of change and people’s ability to identify manipulations. This finding suggests that Delta-E is a more useful measure for local, discrete changes to an image than it is for global image changes, such as applying a filter.

Of course, the whole point of manipulating images is to fool observers, to make them believe that something fake is in fact true. Therefore, it might not be particularly surprising to learn that people find it difficult to spot high quality image manipulations. Yet it is surprising to learn that, even though our subjects never saw the same image more than once, this ability might be dependent on the amount of disruption between the original and manipulated image. The positive relationship between the accurate detection and location of manipulations and Delta-E suggests that it might be possible to develop a metric that allows for a graded prediction about people’s ability to detect and locate image manipulations. The possibility that a metric could be used to predict people’s ability to identify image manipulations is an exciting prospect; however, further research is needed to check that this finding generalizes across a wider variety of images and manipulation types. Our findings suggest that manipulation type and the technique used to create the manipulation, for instance, cloning or scaling, might be less important than the extent to which the change affects the underlying pixel structure of the image. To test this possibility, we next consider the relationship between the Delta-E values and the proportion of (a) correct detection and (b) location responses by the category of manipulation type.

Our findings in Experiments 1 and 2 show that subjects’ ability to detect and locate image manipulations varied by manipulation type, yet, in Experiment 2 the differences were not adequately explained by the plausibility of the manipulation. That is, subjects accurately detected and located more of the addition or subtraction manipulations than the geometry, shadow, or airbrushing manipulations. One possibility is that the five categories of manipulation type introduced different amounts of change between the original and manipulated versions of the images. If so, we might expect these differences in amount of change to help explain the differences in subjects’ detection and localization rates across these categories.

**Fig. 9:**
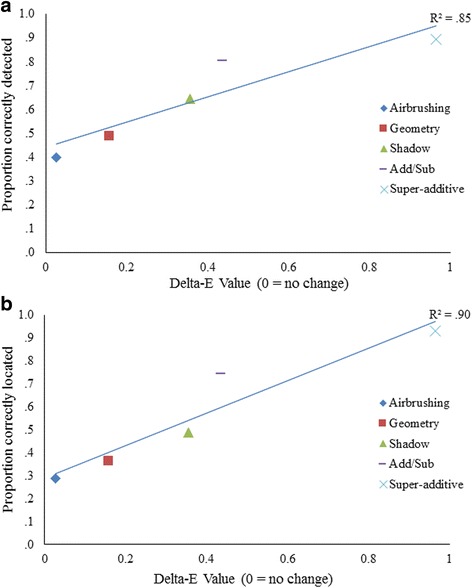
Mean proportion of correctly detected (**a**) and located (**b**) image manipulations by extent of pixel distortion as measured by Delta-E. The graphs show the mean values for each of the five categories of manipulation type

To check this, we calculated the mean proportion of correct detections, localizations, and Delta-E values for each of the five categories of manipulation type. As Fig. 9 shows, there was a positive correlation between the amount of change and the proportion of correct detections (*r*(3) = 0.92, *p* = 0.03) and the proportion of correct localizations (*r*(3) = 0.95, *p* = 0.01). These results suggest that the differences in detection and localization rates across the five manipulation types are better accounted for by the extent of the physical change to the image caused by the manipulation, rather than the plausibility of that manipulation. Yet, given that subjects did not have the opportunity to compare the manipulated and original version of the scene, it is not entirely obvious why amount of change predicts accuracy.

Our results suggest that the amount of change between the original and manipulated versions of an image is an important factor in explaining the detectability and localization of manipulations. Next we considered whether any individual factors are associated with improved ability to detect or locate manipulations.

#### Factors that mediate the ability to detect and locate manipulations

Using GEE analyses, we again explored various factors that might affect people’s ability to detect and locate manipulations. As discussed, we were able to use liberal or stringent criteria for our classification of detection and location accuracy on the manipulated image trials. Accordingly, we ran three models: the first two used the liberal classification for accuracy (and replicated the models we ran in Experiment 1), and the other examined the more stringent classification, DL. As in Experiment 1, for the detection task, we also ran two repeated measures linear regression GEE models to explore the effect of the predictor variables on signal-detection estimates *d'* and *c*. We included the same factors used in the GEE models in Experiment 1. The results of the GEE analyses are shown in Table 5.

**Table 5:** Results of the GEE binary logistic and linear regression models to determine variables that predict accuracy in the detect and locate tasks

Predictor	*B*	*OR [95% CI]*	*p*
Detect (DL and DnL)			
Response time			
Accuracy	0.13	1.14 [1.10, 1.18]	<0.001
*d'*	−0.01	0.99 [0.98, 1.01]	0.40
*c*	0.004	1.00 [0.99, 1.01]	0.42
General belief about percentage of images manipulated = High (71–100%)			
Accuracy	0.16	1.18 [1.02, 1.36]	0.03
*d'*	0.09	1.09 [0.97, 1.23]	0.14
*c*	−0.04	0.96 [0.90, 1.03]	0.25
Gender = Female			
Accuracy	−0.01	0.99 [0.86, 1.15]	0.92
*d'*	−0.03	0.97 [0.86, 1.09]	0.60
*c*	−0.01	0.99 [0.93, 1.06]	0.82
Interest in photography = Interested			
Accuracy	0.17	1.19 [1.02, 1.39]	0.03
*d'*	0.04	1.04 [0.92, 1.18]	0.56
*c*	−0.05	0.95 [0.89, 1.02]	0.18
Frequency of taking photos = Daily/weekly			
Accuracy	−0.01	0.99 [0.84, 1.17]	0.91
*d'*	−0.07	0.93 [0.82, 1.07]	0.31
*c*	−0.05	0.95 [0.88, 1.02]	0.18
Locate (DL and nDL)			
Response time	0.10	1.11 [1.08, 1.14]	<0.001
General belief about percentage of images manipulated = High (71–100%)	−0.01	0.99 [0.87, 1.12]	0.84
Gender = Female	−0.10	0.91 [0.80, 1.03]	0.14
Interest in photography = Interested	0.16	1.17 [1.02, 1.34]	0.02
Frequency of taking photos = Daily/weekly	−0.08	0.92 [0.80, 1.06]	0.27
Detect and locate (DL)			
Response time: detect	0.17	1.19 [1.15, 1.23]	<0.001
Response time: locate	0.13	1.14 [1.11, 1.18]	<0.001
General belief about percentage of images manipulated = High (71–100%)	0.05	1.05 [0.91, 1.20]	0.51
Gender = Female	−0.13	0.88 [0.77, 1.01]	0.07
Interest in photography = Interested	0.20	1.22 [1.06, 1.41]	0.01
Frequency of taking photos = Daily/weekly	−0.09	0.92 [0.78, 1.07]	0.28

B and odds ratios (OR) estimate the degree of change in (a) accuracy on the task (based on the manipulated image trials), (b) *d'*, or (c) *c* associated with one unit change in the independent variable. An odds ratio of 1 indicates no effect of the independent variable on accuracy; values of 1.5, 2.5, and 4.0 are generally considered to reflect small, medium, and large effect sizes, respectively (Rosenthal, 1996). The category order for factors was set to descending to make the reference level 0. The reference groups are: General beliefs about percentage of images manipulated = Low (0–70%), Gender = Male, Interest in photography = Not Interested, Frequency of taking photos = Monthly/yearly/never. For response time (RT) we divided the data into eight equal groups with level 1 representing the slowest RTs (detect ≥47.1 s; locate ≥18.9 s) and level 8 the fastest (detect ≤8.1 s; locate ≤2.7 s). The 14 subjects who chose not to disclose their gender were excluded from these analyses, leaving a total sample of *n* = 645. Because we did not have a fixed number of choices per condition in the location task, we were unable to calculate the degree of change in *d'* or *c* associated with the predictor variables

Using the more liberal accuracy classification, that is, both DL and DnL responses for detection, we found that three factors had an effect on likelihood to respond correctly: response time, general beliefs about the prevalence of photo manipulation, and interest in photography. As in Experiment 1, faster responses were more likely to be correct than slower responses. Also replicating the finding in Experiment 1, those who believe a greater percentage of photos are digitally manipulated were slightly more likely to correctly identify manipulated photos than those who believe a lower percentage of photos are digitally manipulated. Additionally, in Experiment 2, those interested in photography were slightly more likely to identify image manipulations correctly than those who are not interested in photography. For the location task, using the more liberal accuracy classification, that is, both DL and nDL responses, we found that two factors had an effect on likelihood to respond correctly. Again there was an effect of response time: In the location task, faster responses were more likely to be correct than slower responses. Also those with an interest in photography were slightly more likely to correctly locate the manipulation within the photo than those without an interest. Next we considered whether any factors affected our more stringent accuracy classification, that is, being correct on both the detection and location tasks (DL). The results revealed an effect for two factors on likelihood to respond correctly. Specifically, there was an effect of response time with shorter response times being associated with greater accuracy. There was also an effect of interest in photography, with those interested more likely to correctly make DL responses than those not interested.

Our GEE models in both Experiments 1 and 2 revealed that shorter response times were linked with more correct responses on both tasks. As in Experiment 1, this association might be explained by several models of perceptual decision making; however, determining which of these models best accounts for our data is beyond the scope of the current paper.

## General discussion

In two separate experiments we have shown, for the first time, that people’s ability to detect manipulated photos of real-world scenes is extremely limited. Considering the prevalence of manipulated images in the media, on social networking sites, and in other domains, our findings warrant concern about the extent to which people may be frequently fooled in their daily lives. Furthermore, we did not find any strong evidence to suggest that individual factors, such as having an interest in photography or beliefs about the extent of image manipulation in society, are associated with improved ability to detect or locate manipulations.

Recall that we looked at two categories of manipulations—implausible and plausible—and we predicted that people would perform better on implausible manipulations because these scenes provide additional evidence that people can use to determine if a photo has been manipulated. Yet the story was not so simple. In Experiment 1, subjects correctly detected more of the implausible photo manipulations than the plausible photo manipulations, but in Experiment 2, the opposite was true. Further, even when subjects correctly identified the implausible photo manipulations, they did not necessarily go on to accurately locate the manipulation. It is clear that people find it difficult to detect and locate manipulations in real-world photos, regardless of whether those manipulations lead to physically plausible or implausible scenes.

Research in the vision science literature may help to account for these findings. We know that people might have a simplified understanding of the physics in our world (Cavanagh, 2005; Mamassian, 2008). Studies have shown, for instance, that the human visual system is relatively insensitive to the physically impossible cast shadows created by inconsistent lighting in a scene (Ostrovsky, Cavanagh, & Sinha, 2005). It is not necessarily the case that people ignore shadows altogether, but rather that the visual system processes shadows rapidly and uses them only as a generic cue. Put simply, as long as the shadow is roughly correct then we accept it as being authentic (Bonfiglioli, Pavani, & Castiello, 2004; Ostrovsky et al., 2005; Rensink & Cavanagh, 2004). Similarly, people use shortcuts to interpret geometrical aspects of a scene; if the geometry is close enough to people’s expectation, then it is accepted as accurate (Bex, 2010; Howe & Purves, 2005; Mamassian, 2008). Furthermore, the change blindness literature also highlights people’s insensitivity to shadow information. Research has shown that people are slower to detect changes to cast shadows than changes to objects (Wright, 2005), even when the shadow changes affect the overall meaning of the scene (Ehinger, Allen, & Wolfe, 2016). It follows, then, that when trying to distinguish between real and manipulated images, our subjects do not seem to have capitalized on the evidence in the implausible manipulation photos to determine whether they were authentic. It remains to be determined whether it is possible to train people to make use of physically implausible inconsistencies; perhaps one possibility would entail “teaching” the visual system to make full use of physical properties of the world as opposed to automatically simplifying them.

Although the plausibility of a manipulation might not be so important when it comes to detecting manipulated images, we found that the extent to which the manipulation disrupts the underlying structure of the pixels might be important. Indeed, we found a positive correlation between the image metric (Delta-E) we used to measure the difference between our original and manipulated photos and the likelihood that the photo was correctly classified as manipulated. In other words, the manipulations that created the most change in the underlying pixel values of the photo were most likely to be correctly classified as manipulated. Of course, from the perspective of signal detection theory, it follows that adding greater signal results in greater detection of that signal (Green & Swets, 1966; Wilken & Ma, 2004).

Although this might seem intuitive, recall that our subjects never saw the same scene more than once. That is, they never saw the non-manipulated versions of any of the manipulated photos that they were shown; despite this, their ability to detect the manipulated photos was related to the extent of change in the pixels. It seems possible that our subjects might have been able to compare the manipulated photo with their expectations about what the scene “should” look like in terms of scene statistics. In doing this, subjects might have found the manipulated photos with less change, and thus smaller Delta-E values, were more similar to their prior expectations of what the world looks like—resulting in those photos being incorrectly accepted as authentic more often. At the same time, the manipulated photos with more change, and thus larger Delta-E values, may have been more difficult to match to a prior expectation—resulting in these photos more often being correctly identified as manipulated. It seems that this difference in ease of finding a match to prior knowledge and expectation for the manipulated photo helped subjects to make an accurate decision. If this is the case, then one might speculate that it could be possible to develop a metric that will predict people’s ability to detect and locate manipulations of real-world scenes. A future investigation using a wider range of stimuli where subjects see more than one of each manipulation type might consider whether there is an interaction between Delta-E and manipulation type.

On a different note, our research highlights a potential opportunity to improve people’s ability to spot manipulations. In Experiment 2, we were able to compare subjects’ ability on the two tasks: detection and location. We were surprised to find that subjects performed better on the location task than on the detection task. Although this is an interesting finding, the reason for it is not immediately apparent. One possibility is that these two tasks might encourage subjects to adopt different strategies and that subjects are better at the more direct task of locating manipulations than the generic one of detecting whether a photo has been manipulated or not.

Our research provides a first look at people’s ability to detect and locate manipulations of real-world images. A strength of the current method—applying each of the five different manipulation types to the same image—is that we know the differences in subjects’ performance is owing to the manipulation itself rather than the specific image. A drawback, however, is that the difficulty of finding or generating a set of suitable images that allowed all of the manipulation types to be applied reduced the total number of photos that could be tested to some degree. Although, ideally, future work might extend the range of images tested, we nonetheless note the close consistency in results that we obtained across the two different and independent image sets used in Experiments 1 and 2.

Future research might also investigate potential ways to improve people’s ability to spot manipulated photos. However, our findings suggest that this is not going to be a straightforward task. We did not find any strong evidence to suggest there are individual factors that improve people’s ability to detect or locate manipulations. That said, our findings do highlight various possibilities that warrant further consideration, such as training people to make better use of the physical laws of the world, varying how long people have to judge the veracity of a photo, and encouraging a more careful and considered approach to detecting manipulations. What our findings have shown is that a more careful search of a scene, at the very least, may encourage people to be skeptical about the veracity of photos. Of course, increased skepticism is not perfect because it comes with an associated cost: a loss of faith in authentic photos. Yet, until we know more about how to improve people’s ability to distinguish between real and fake photos, a skeptical approach might be wise, especially in contexts such as law, scientific publication, and photojournalism where even a small manipulation can have ethically significant consequences.

But what should we be skeptical about? Are some changes acceptable and others not? Should the context of the manipulation be taken into account? Though we are unable to answer these complex questions here, we can offer some points for thought. Although it is true that all image manipulations are to some extent deceptive, not all manipulations are *intentionally* deceptive. This distinction is an important one and raises the possibility that people do not set out to detect all image manipulations but instead are primarily concerned about forgeries that have been created with the intention to deceive the viewer. Of course, people might expect that all images provided as evidence, for instance news images, to have been subjected to rigorous validation processes. It is unlikely, however, that people set themselves the same standard for detecting manipulation in every day contexts. Perhaps more important than being able to identify all instances of manipulation, people are most concerned about the extent to which they can trust the message conveyed from the image. Although this poses an interesting question, our results suggest that people might struggle to detect image manipulations based on either of these definitions. In the current research, not only did subjects find it difficult to accurately locate the specific aspects of the image that had been altered, they also found it difficult to distinguish original, truthful photos from manipulated, untruthful ones.

In light of the findings presented in this paper, it is not surprising that World Press Photo have introduced a computerized photo-verification test to their annual photo contest. But at the end of the day, this is only a competition. What do our findings mean for other contexts in which an incorrect decision about the veracity of a photo can have devastating consequences? Essentially, our results suggest that guidelines and policies governing the acceptable standards for the use of photos, for example, in legal and media domains, should be updated to reflect the unique challenges of photography in the digital age. We recommend that this is done soon, and that psychological scientists work together with digital forensic experts and relevant end-users to ensure that such policies are built on sound empirical research.

## Conclusions

The growing sophistication of photo-editing tools means that nearly anyone can make a convincing forgery. Despite the prevalence of manipulated photos in our everyday lives, there is a lack of research directly investigating the applied question of people’s ability to detect photo forgeries. Across two experiments, we found that people have an extremely limited ability to detect and locate manipulations of real-world scenes. Our results in Experiment 1 offer some support to the suggestion that people are better able to identify physically implausible changes than physically plausible ones. But we did not replicate this finding in Experiment 2; instead, our results indicate that the amount of change is more important than the plausibility of the change when it comes to detecting and localizing manipulations. Furthermore, we did not find any strong evidence to suggest individual factors are associated with improved ability to detect or locate manipulations. These findings offer an important first step in understanding people’s ability to identify photo forgeries, and although our results indicate that it might not be an easy task, future research should look to investigate potential ways to improve this ability. Moreover, our results highlight the need to bring current guidelines and policies governing the acceptable standards for the use of photos into the digital age.

## Additional files

Additional file 1: Examples of the images used in Experiments 1 and 2. (PDF 5902 kb)

Additional file 2: Details of the saliency analyses for the stimuli used in Experiments 1 and 2. (DOCX 333 kb)

## Abbreviations

CI: Confidence interval
GEE: Generalized Estimating Equations
JPEG: Joint Photographic Experts Group
OR: Odds ratio
PNG: Portable Network Graphics
RT: Response time

## Footnotes

1 In Experiment 2, subjects attempted to localize the manipulation regardless of their response in the detection task.

2 We report 95% confidence intervals to provide an estimate of the size of the effect—in 95% of cases, the population mean will fall within this range of values (Cumming, 2012).

3 One limitation of the Delta-E measure is that a global change to an image, for instance adjusting the brightness of the entire image, would result in a high Delta-E value, yet such a change is likely to be difficult to detect. That said, in our research we are only concerned with local image changes and therefore Delta-E provides a useful measure.

4 This is based on a two-tailed test, given that we would predict that detection rates would increase with the amount of change, we might consider a one-tailed test to be appropriate. With a one-tailed test, the relationship between Delta-E and the proportion of photos correctly detected as manipulated would be significant at the 0.035 level.
