# Supplementary material for: Can people identify original and manipulated photos of real-world scenes?
Source: Cogn Res Princ Implic. 2017 Jul 18;2:30. doi: 10.1186/s41235-017-0067-2 (PMC5514174; doi:10.1186/s41235-017-0067-2)

Experiment 1.
Image C.
Original.

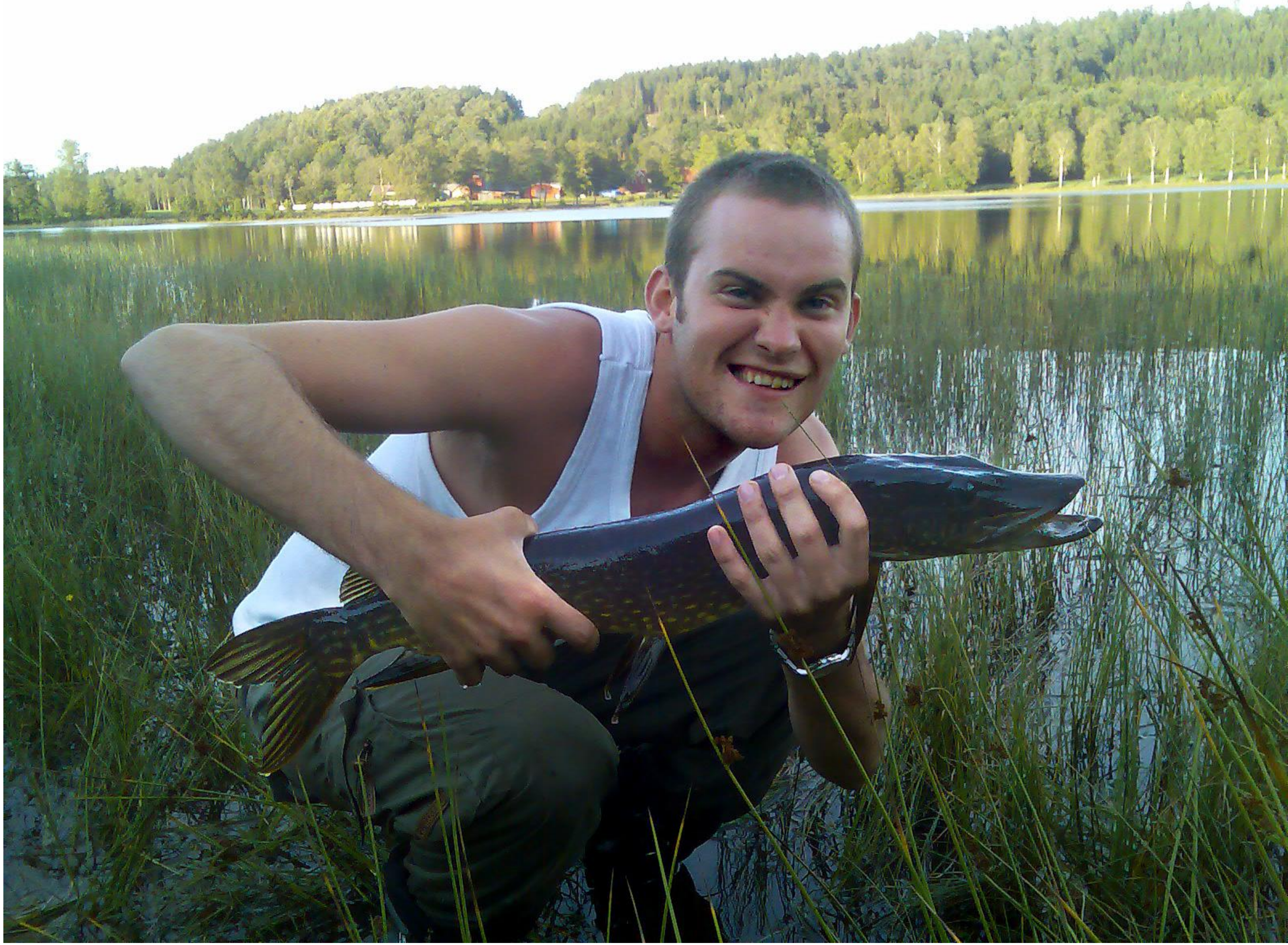

Photo credit:
https://www.flickr.com/photos/9124789@N06/822571846 by Rasmus Zwickson, used under CC BY 2.0

Experiment 1.
Image C.
Airbrushing—brightening of face and eyes, removal of blemishes on the chin, whitening of teeth, and removal of blade of grass covering part of the man's face

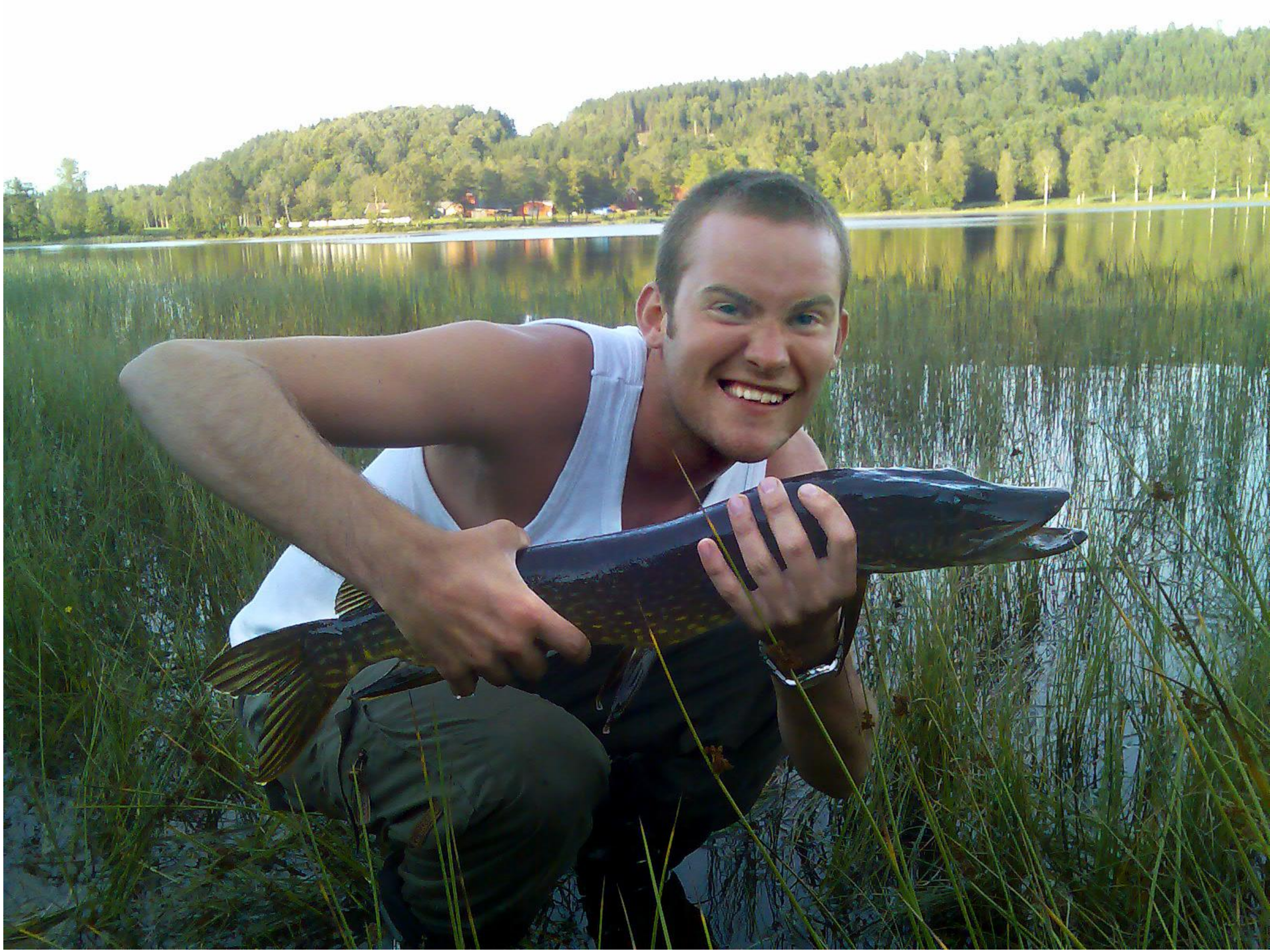

Photo credit: https://www.flickr.com/photos/9124789@N06/822571846 by Rasmus Zwickson, used under CC BY 2.0 / Airbrushing manipulation applied to original

Experiment 1.
Image C.
Addition/Subtraction—
addition of boat on the
water

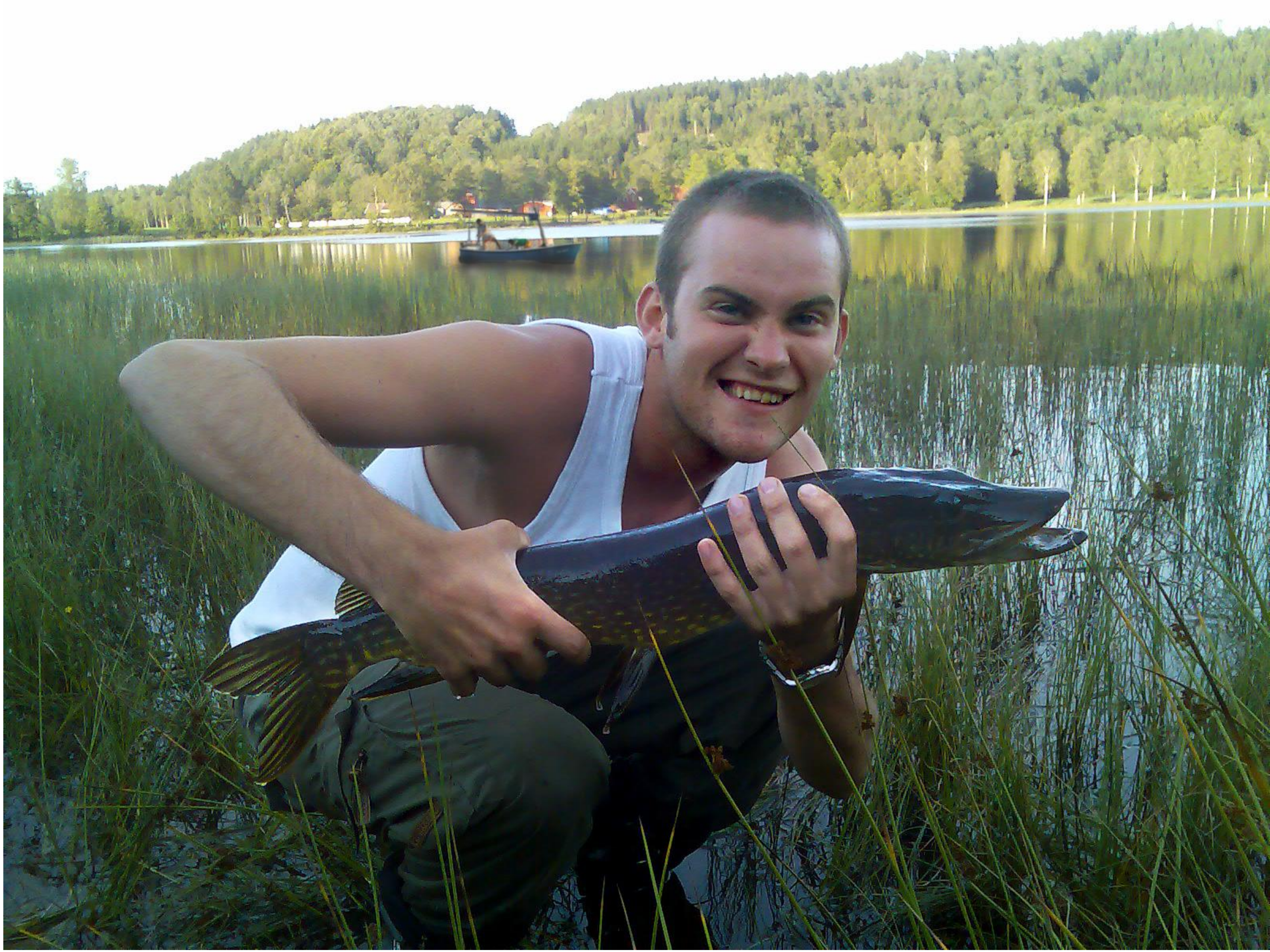

Photo credit:
https://www.flickr.com/photos/9124789@N06/822571846 by Rasmus Zwickson, used under CC BY 2.0 / Addition/Subtraction manipulation applied to original

Experiment 1.
Image C.
Geometry—trees on the left are sheered at an angle so that they appear inconsistent with other trees in the background

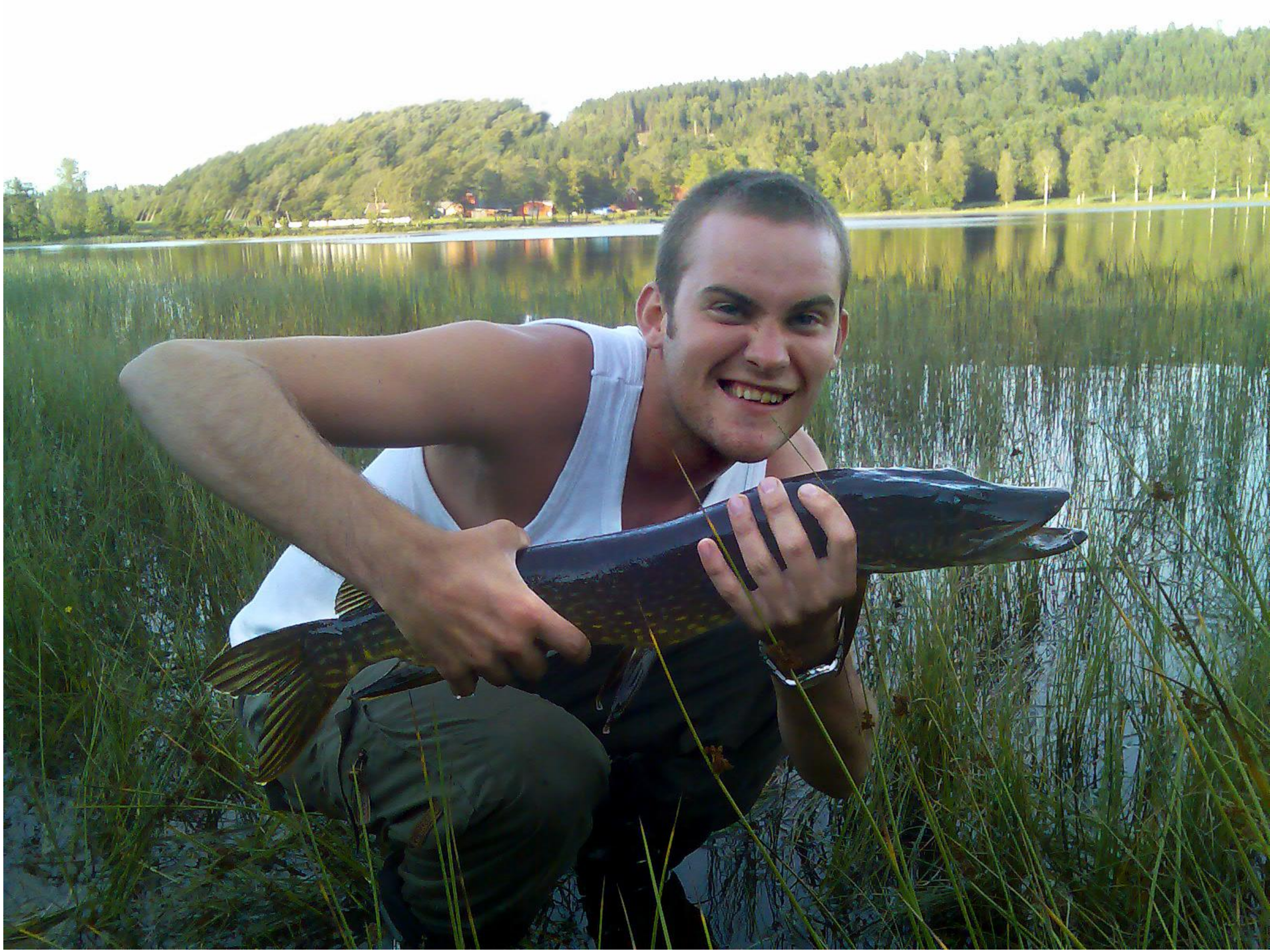

Photo credit:
https://www.flickr.com/photos/9124789@N06/822571846 by Rasmus Zwickson, used under CC BY 2.0 / Geometry manipulation applied to original

Experiment 1.
Image C.
Shadow—removal of the shadows in the trees on the right but the shadows still appear in the trees' reflection in the water

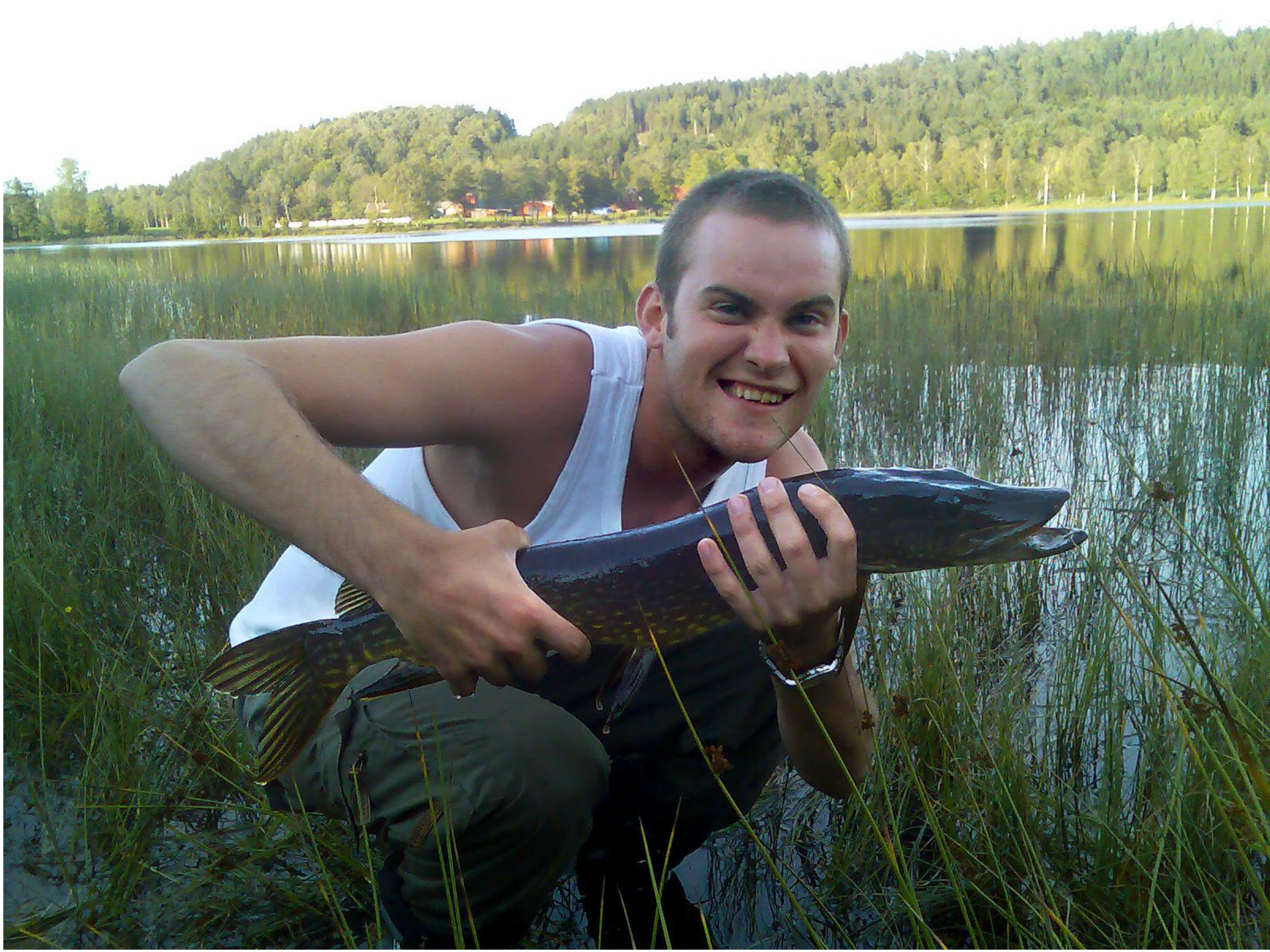

Photo credit:
https://www.flickr.com/photos/9124789@N06/822571846 by Rasmus Zwickson, used under CC BY 2.0 / Shadow manipulation applied to original

Experiment 1.
Image C.
Super-Additive—
combination of all
previously described
manipulations

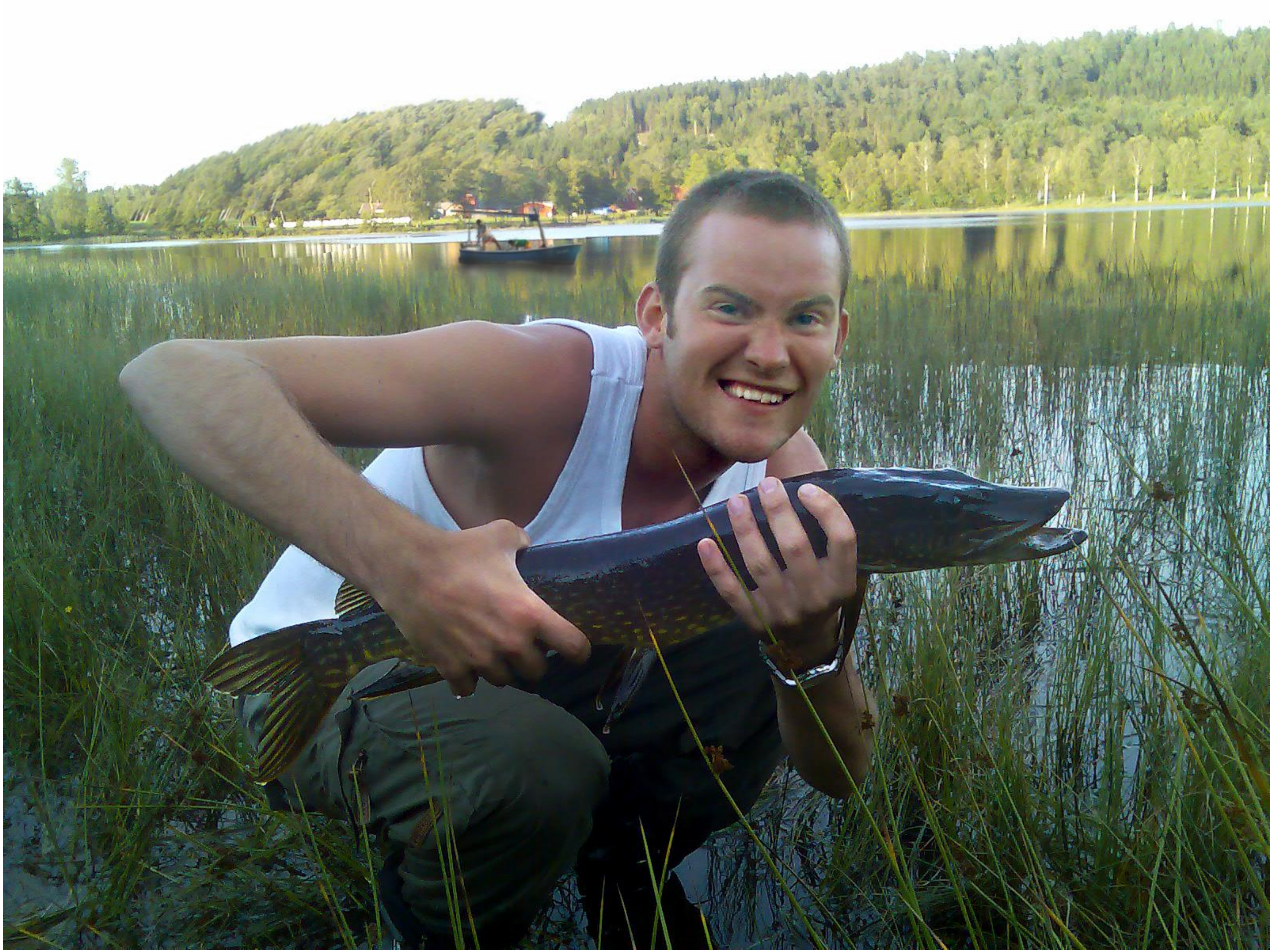

Photo credit:
https://www.flickr.com/photos/9124789@N06/822571846 by Rasmus Zwickson, used under CC BY 2.0 / Super-Additive manipulation applied to original

Experiment 1.
Image F.
Original.

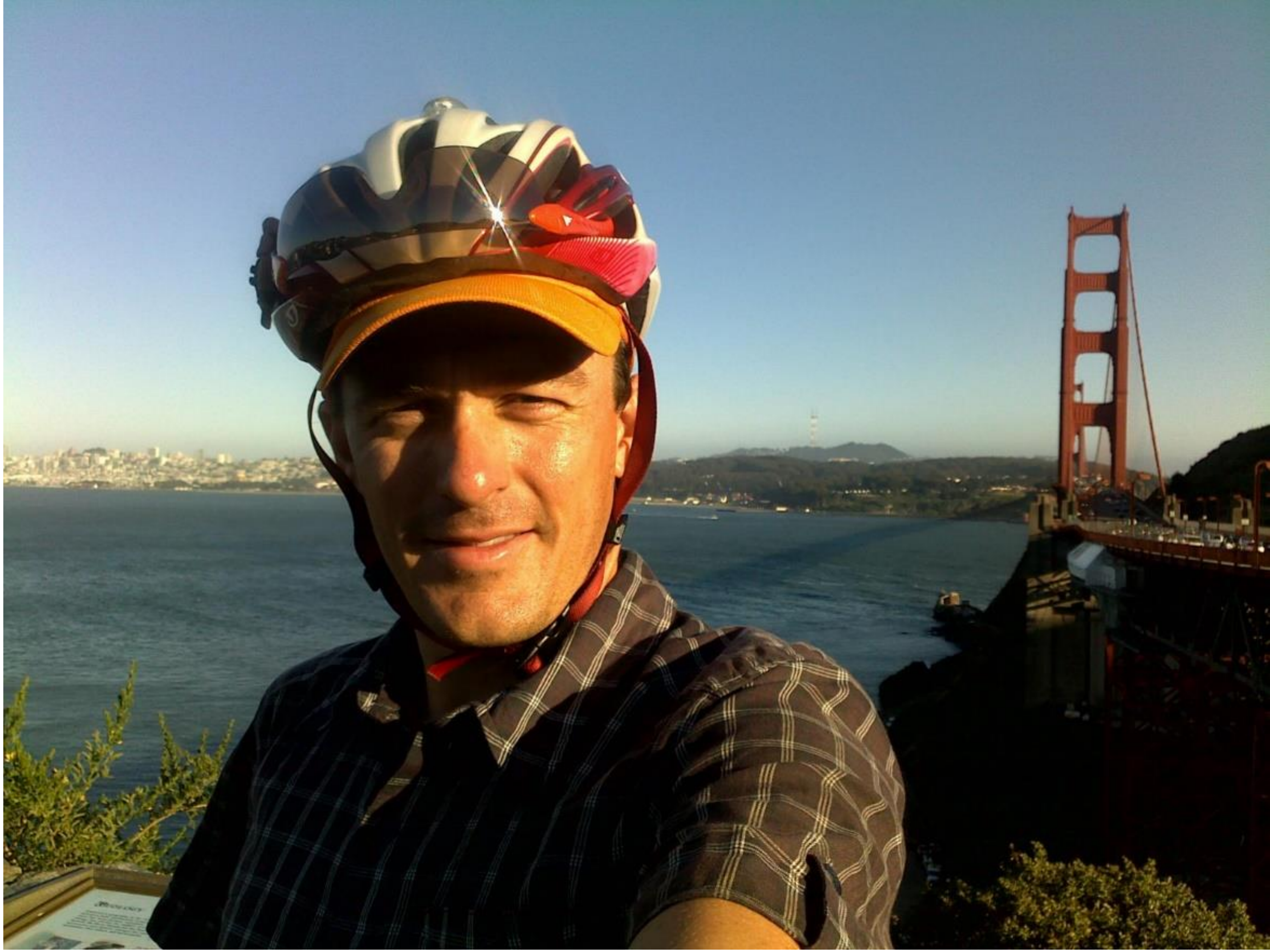

Photo credit:
https://commons.wikimedia.org/wiki/File:Vin_Cox_during_his_Guinness_World_Record_breaking_ride_around_the_world..jpg by Vin Cox, used under CC BY-SA 3.0

Experiment 1.
Image F.
Airbrushing—removal of sweat on the nose, cheeks and chin, and removal of wrinkles around the eyes

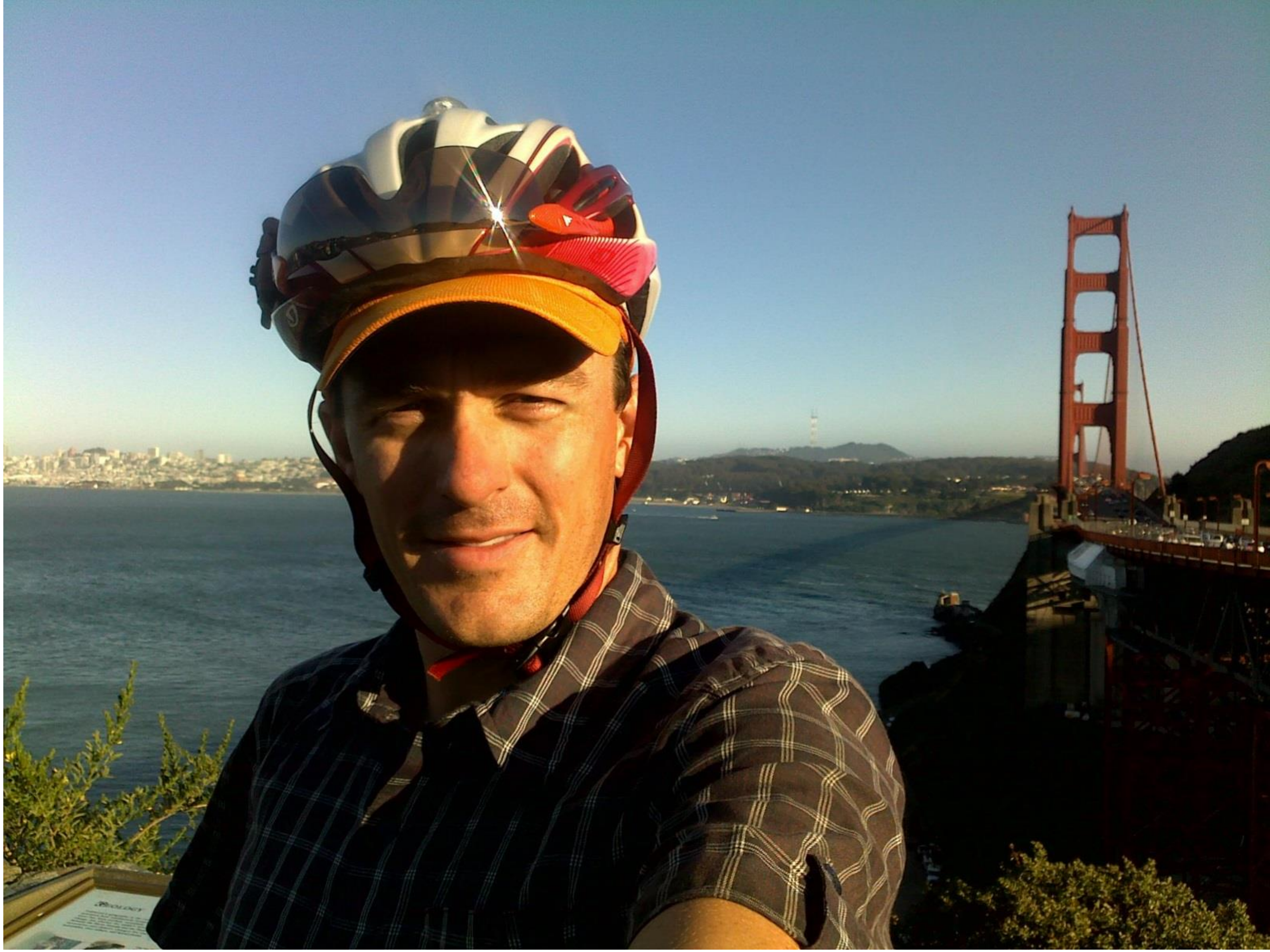

Photo credit: https://commons.wikimedia.org/wiki/File:Vin_Cox_during_his_Guinness_World_Record_breaking_ride_around_the_world..jpg by Vin Cox, used under CC BY-SA 3.0 / Airbrushing manipulation applied to original. Image F Airbrushing is licensed under CC BY-SA 4.0 by Sophie Nightingale.

Experiment 1.
Image F.
Addition/ Subtraction—
two links between the
columns of the tower of
the suspension bridge
removed

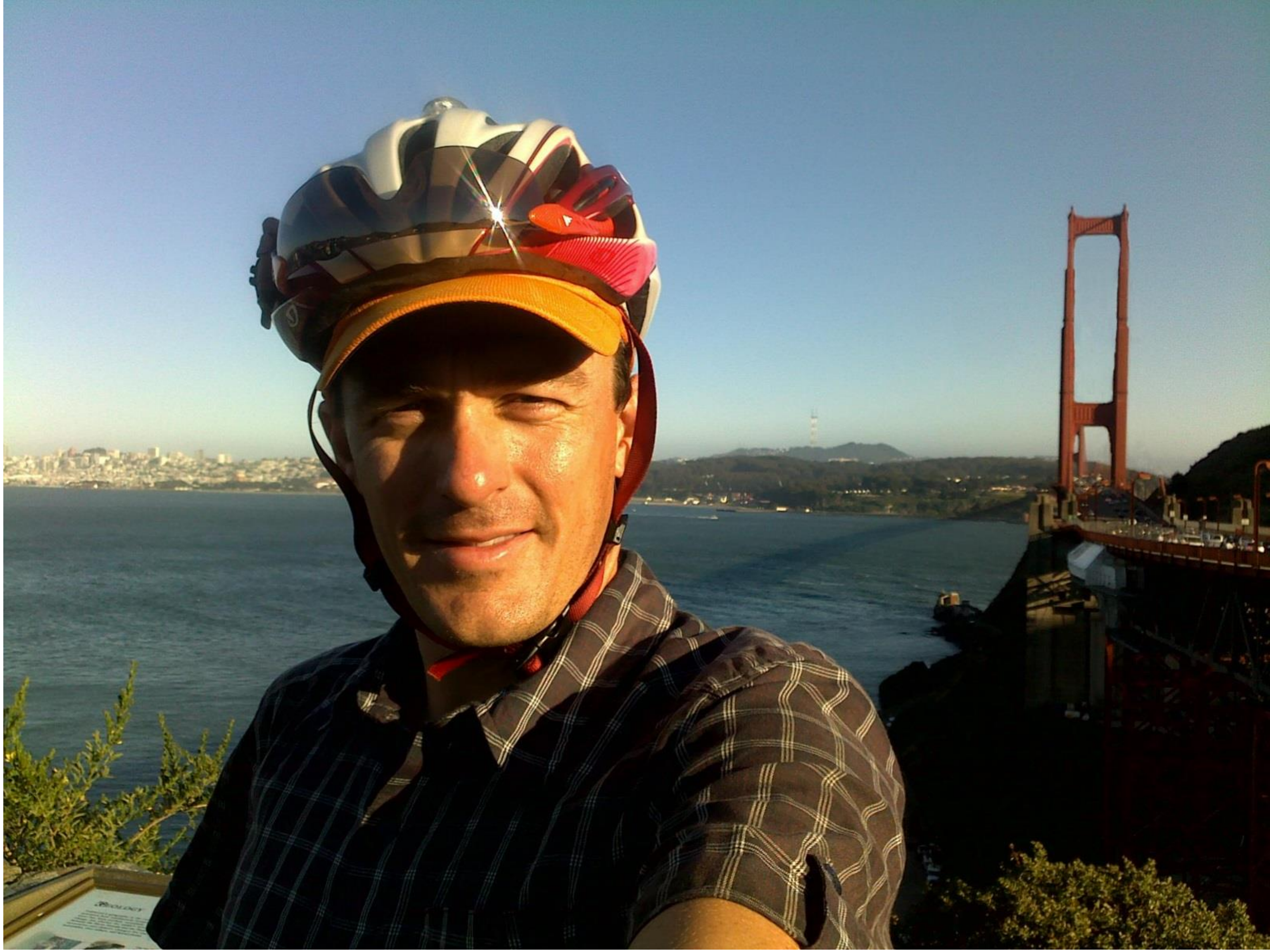

Photo credit:
https://commons.wikimedia.org/wiki/File:Vin_Cox_during_his_Guinness_World_Record_breaking_ride_around_the_world..jpg by Vin Cox, used under CC BY-SA 3.0 / Addition/Subtraction manipulation applied to original. Image F Addition/Subtraction is licensed under CC BY-SA 4.0 by Sophie Nightingale.

Experiment 1.
Image F.
Geometry—top of the bridge is sheered at an angle inconsistent with the rest of the bridge

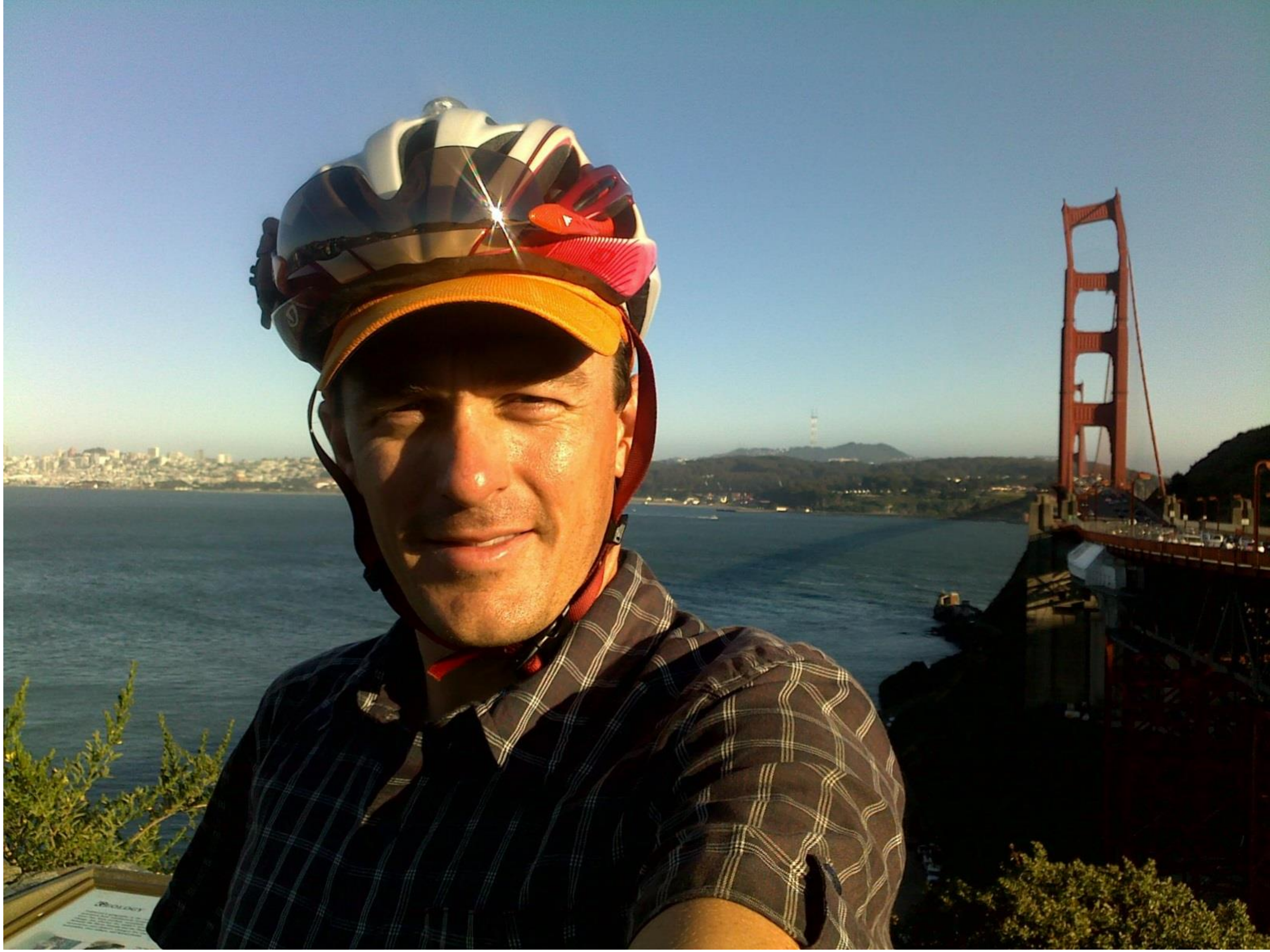

Photo credit: https://commons.wikimedia.org/wiki/File:Vin_Cox_during_his_Guinness_World_Record_breaking_ride_around_the_world..jpg by Vin Cox, used under CC BY-SA 3.0 / Geometry manipulation applied to original. Image F Geometry is licensed under CC BY-SA 4.0 by Sophie Nightingale.

Experiment 1.
Image F.
Shadow—face is flipped horizontally so that the light is on the wrong side of the face compared with lighting in the rest of the scene

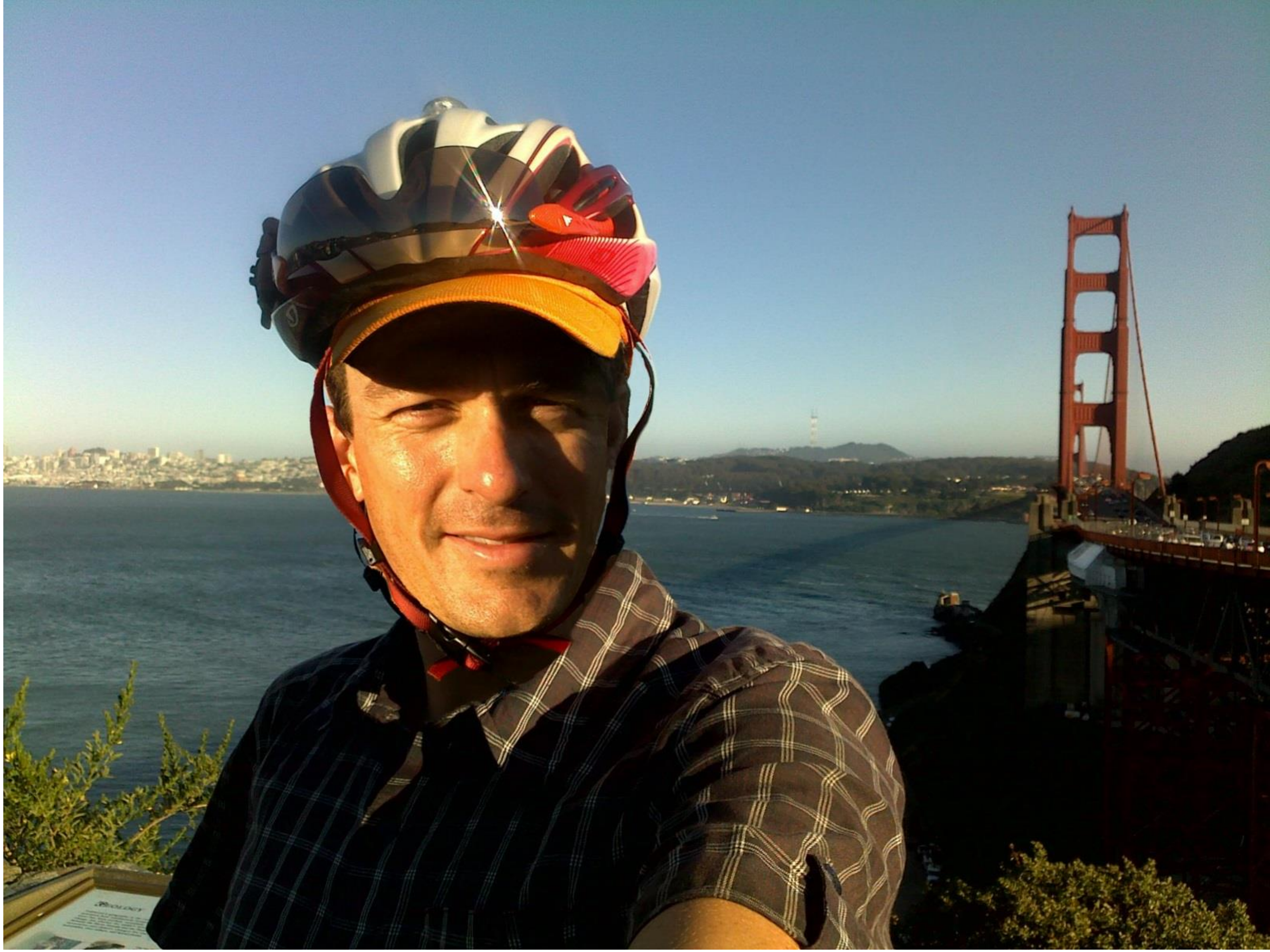

Photo credit:
https://commons.wikimedia.org/wiki/File:Vin_Cox_during_his_Guinness_World_Record_breaking_ride_around_the_world..jpg by Vin Cox, used under CC BY-SA 3.0 / Shadow manipulation applied to original. Image F Shadow is licensed under CC BY-SA 4.0 by Sophie Nightingale.

Experiment 1.
Image F.
Super-Additive—
combination of all
previously described
manipulations

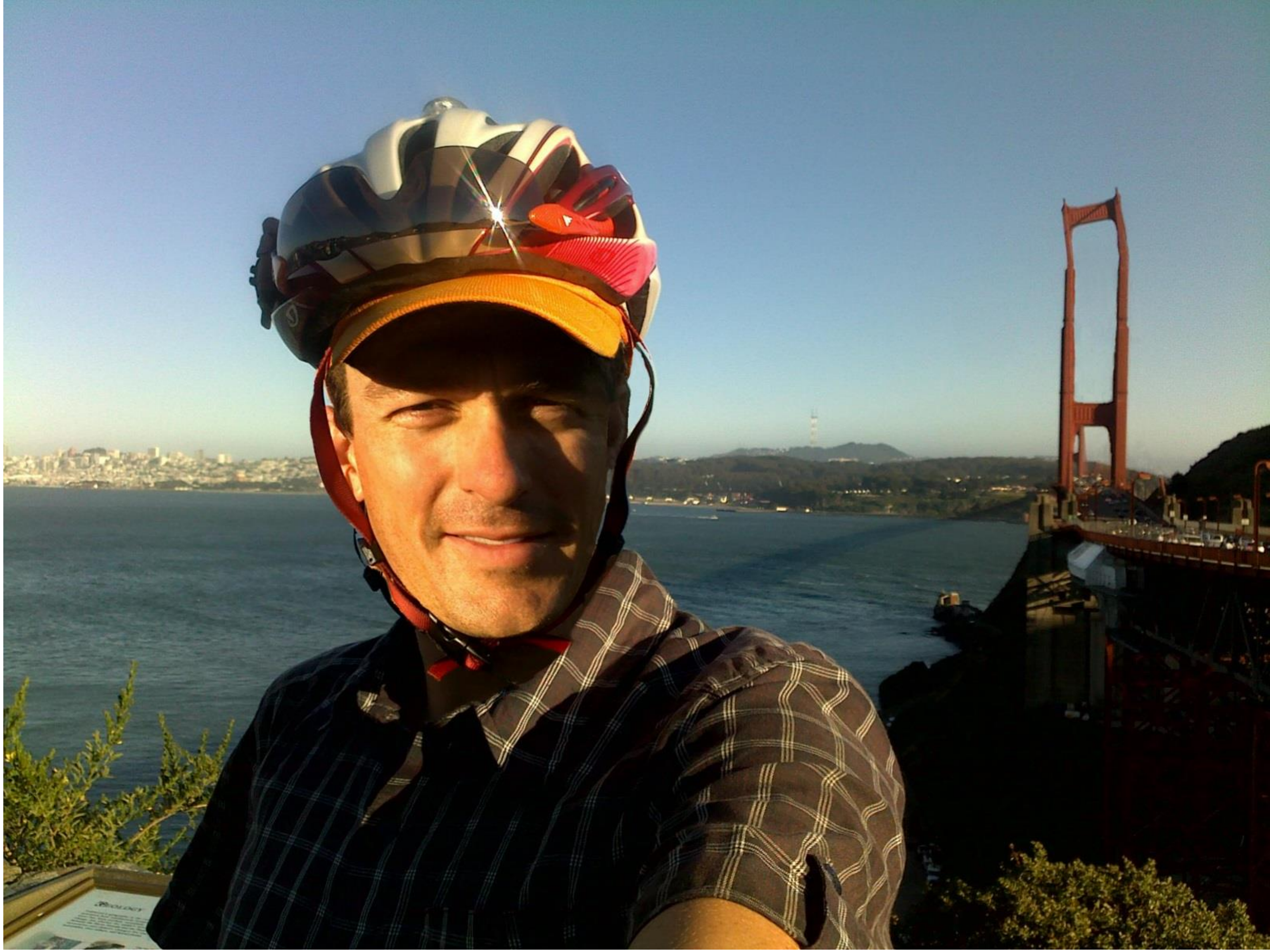

Photo credit:
https://commons.wikimedia.org/wiki/File:Vin_Cox_during_his_Guinness_World_Record_breaking_ride_around_the_world..jpg by Vin Cox, used under CC BY-SA 3.0 / Super-Additive manipulation applied to original. Image F Super-Additive is licensed under CC BY-SA 4.0 by Sophie Nightingale.

Experiment 2.
Image B.
Original.

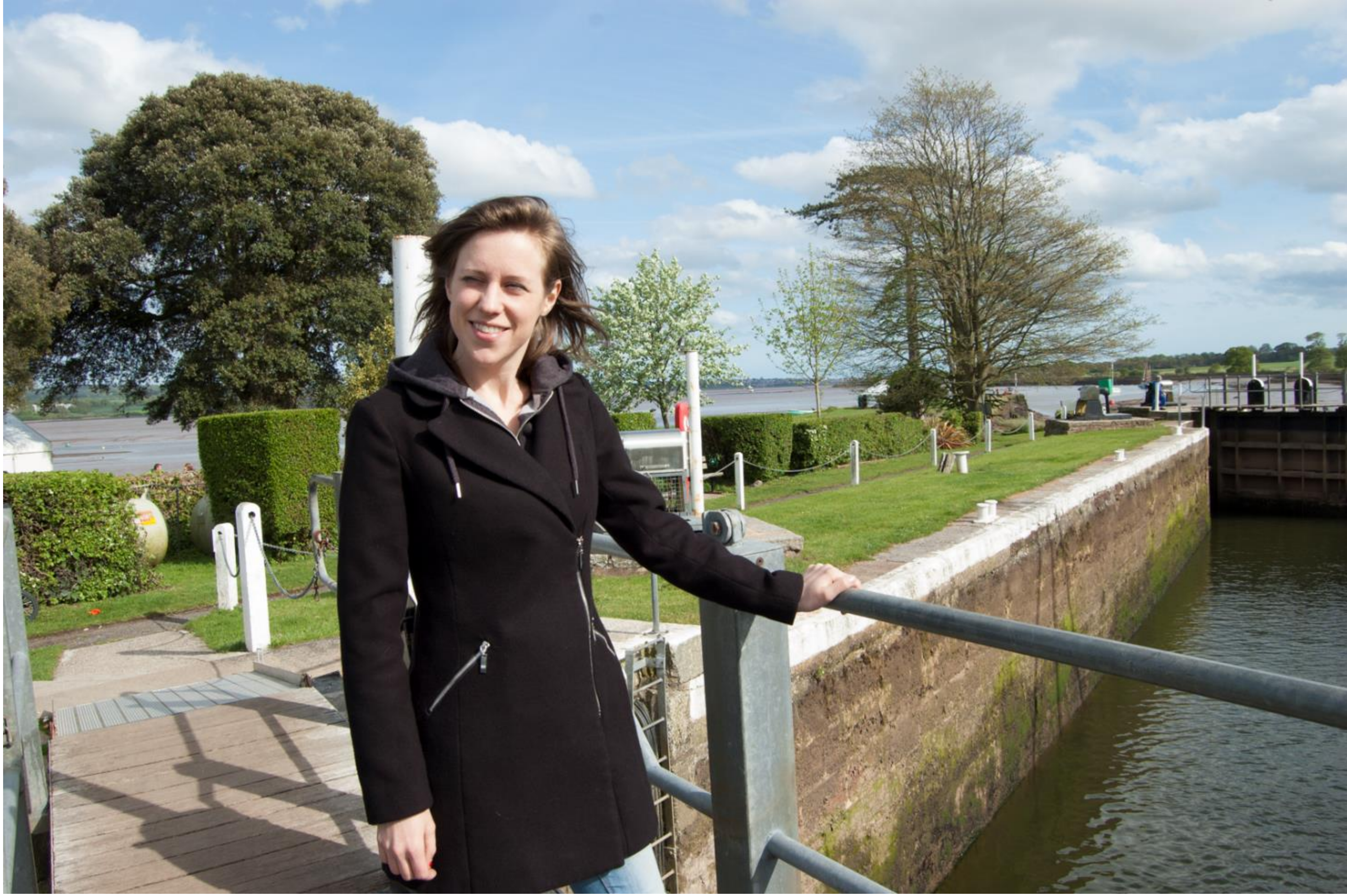

Experiment 2. Image B. Airbrushing—removal of blemishes on the forehead, cheeks, and chin, removal of shadows around the eyes, whitened teeth, rounded face shape, and added volume to hair

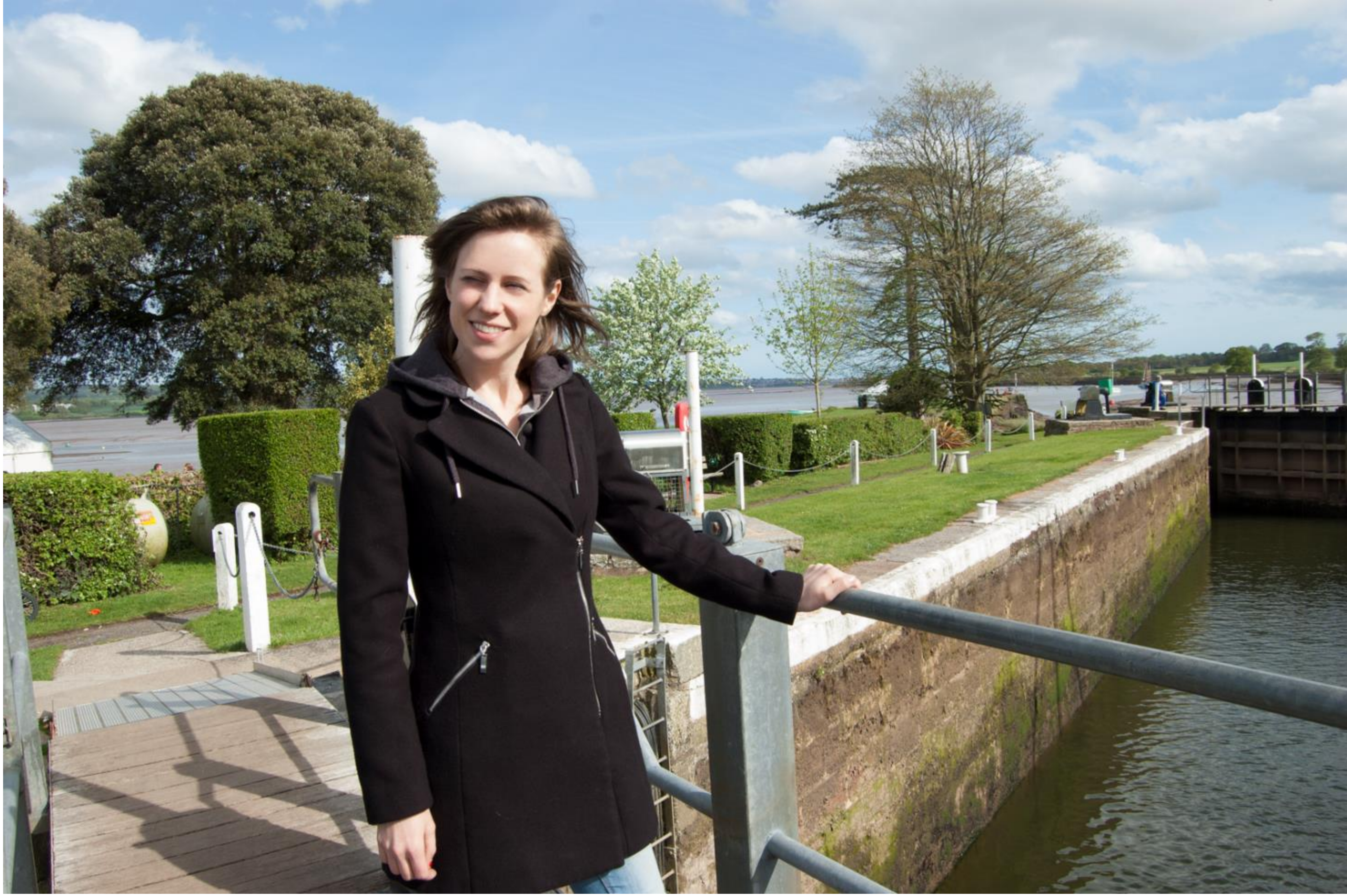

Experiment 2.
Image B.
Addition/
Subtraction—
addition of
water pipe to
the lock wall

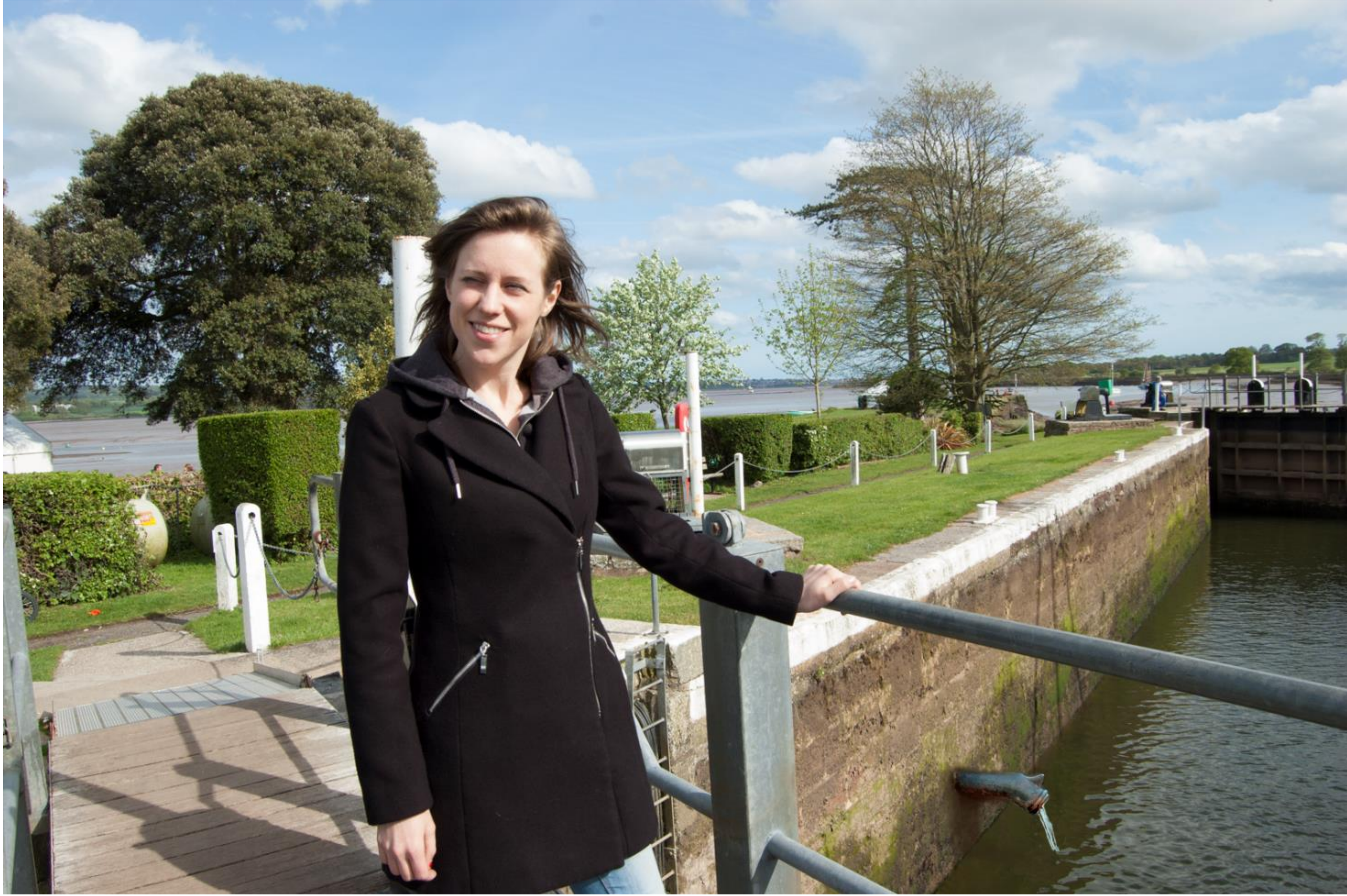

Experiment 2. Image B. Geometry—two columns between the chain railings moved in centre of the scene leaving the railing hanging freely in the air

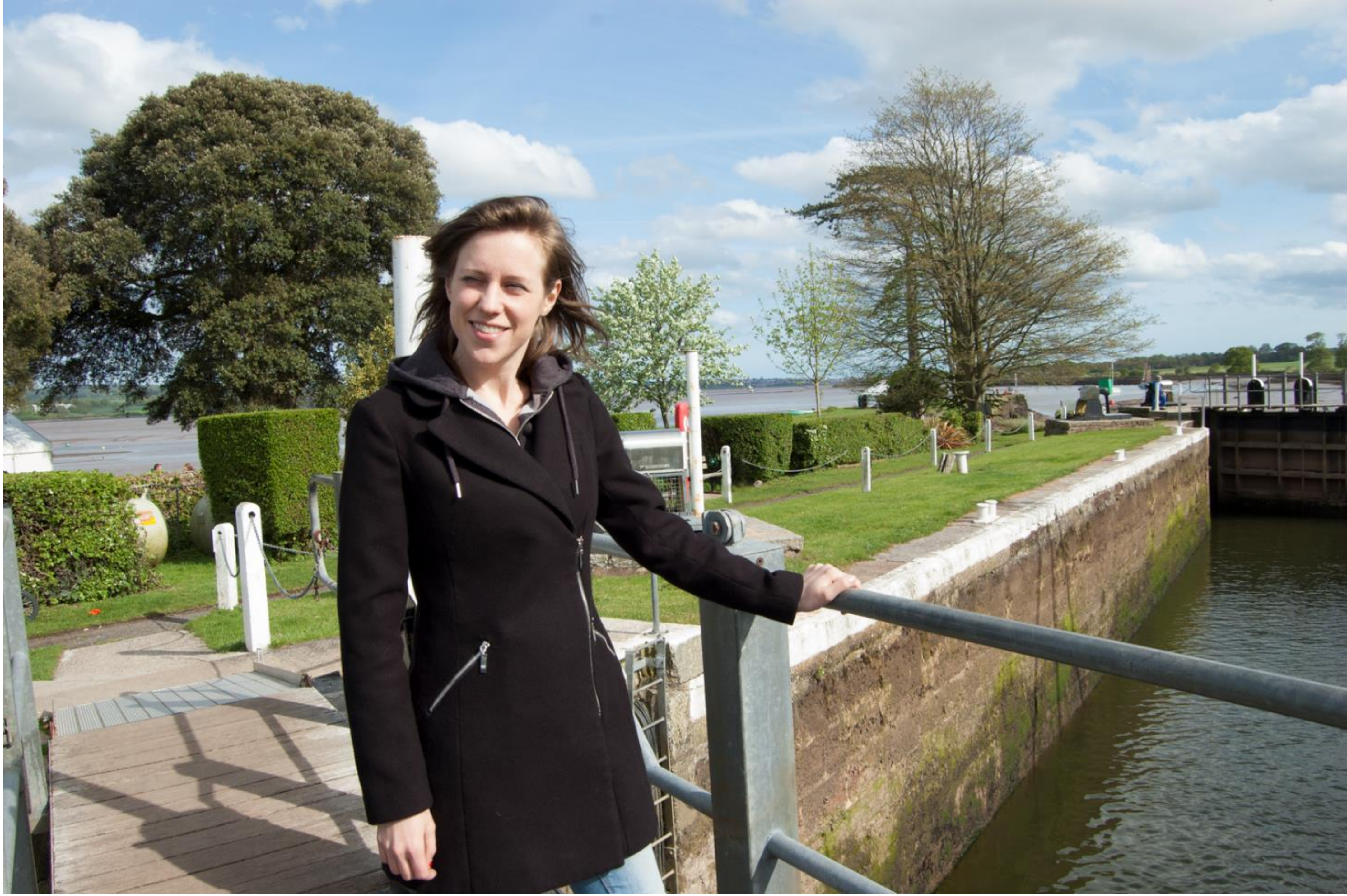

Experiment 2. Image B. Shadow—elongated shadows of the white columns to left of the scene and the inclusion of shadows for extra railings that do not exist on the object

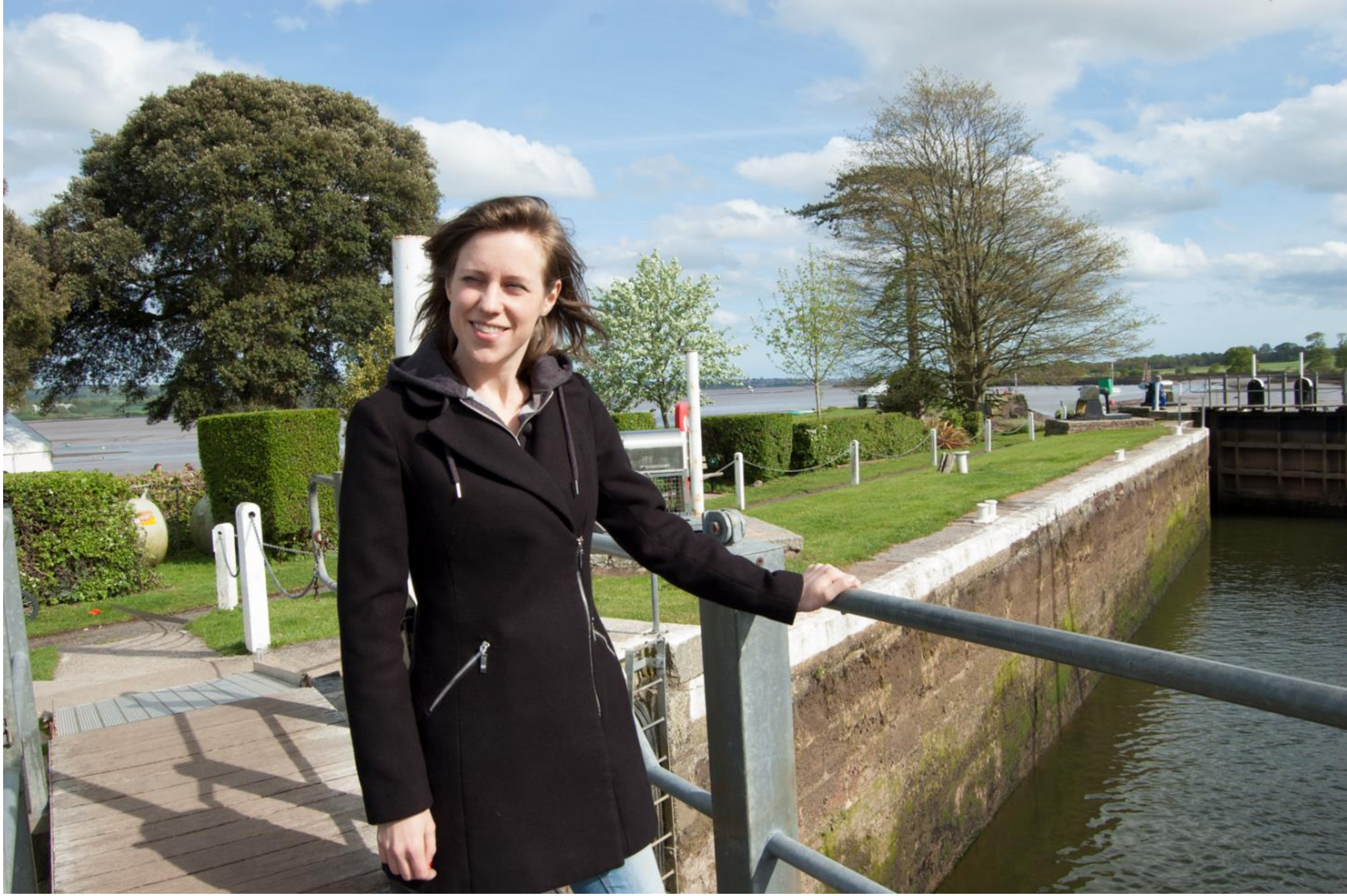

Experiment 2.
Image B.
Super-
Additive—
combination of
all previously
described
manipulations

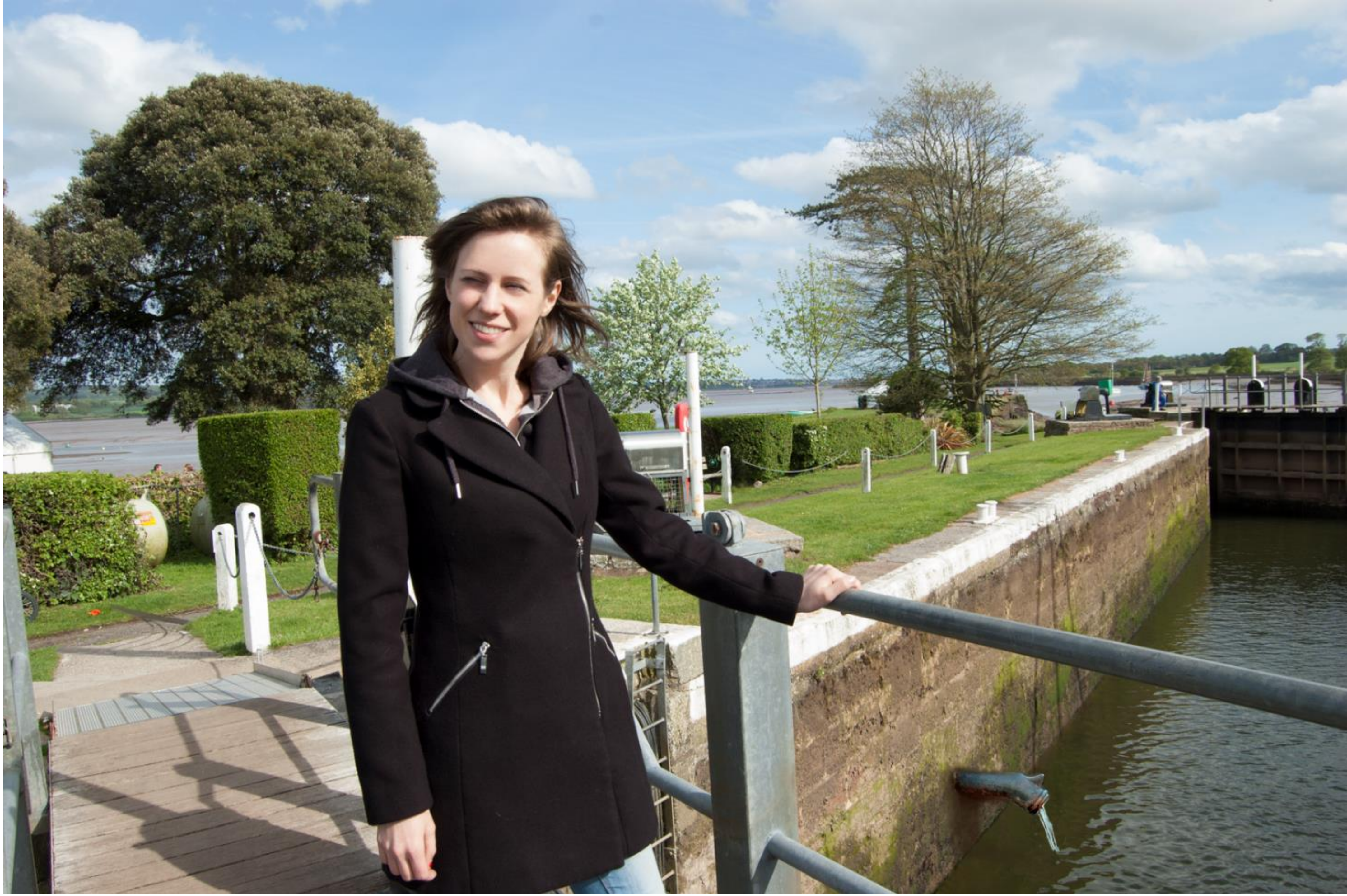

Experiment 2.
Image D.
Original.

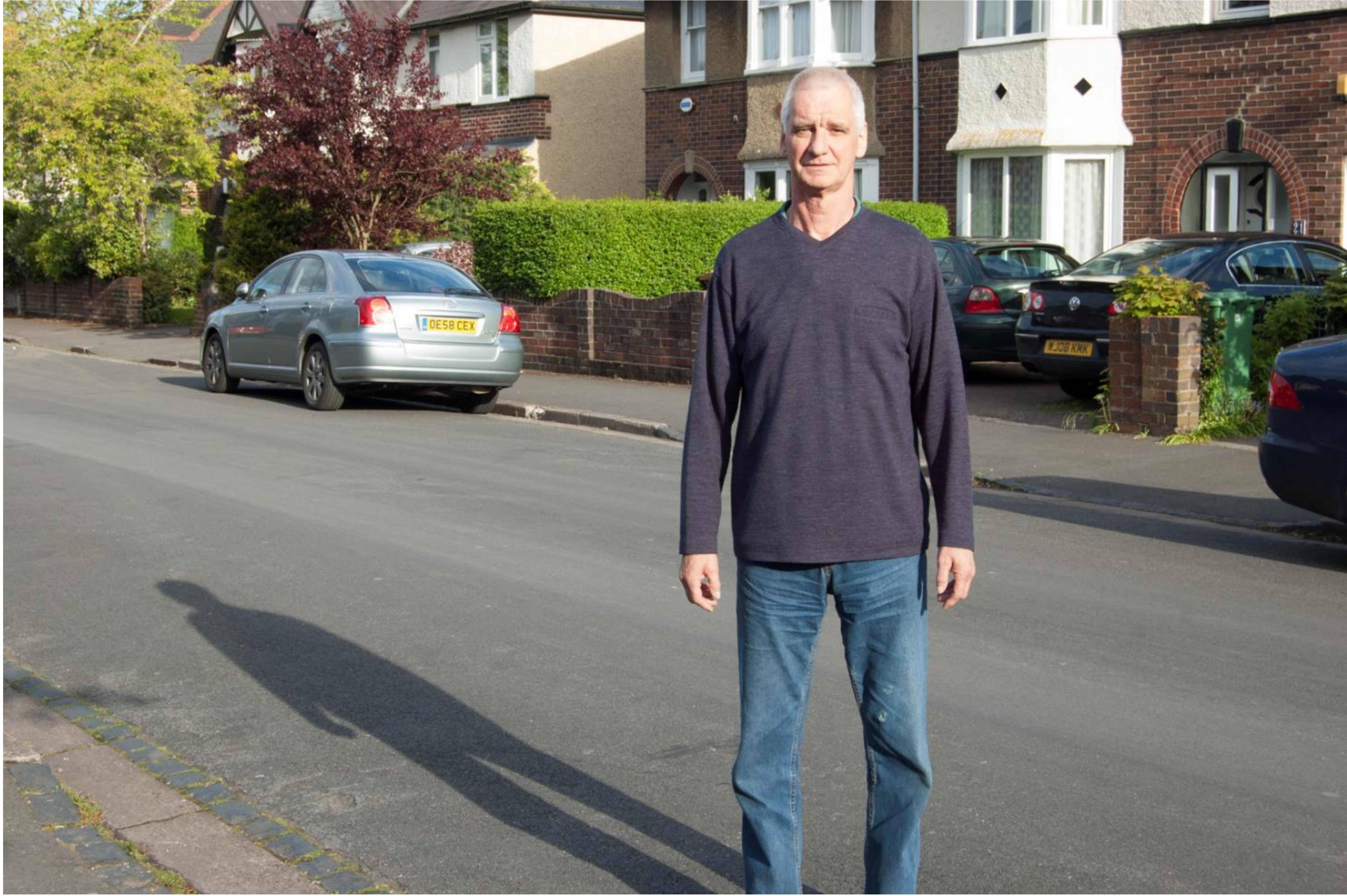

Experiment 2. Image D. Airbrushing—removal of wrinkles on the forehead and neck, removal of blemish from left cheek and top of lip, and filling in receding hairline

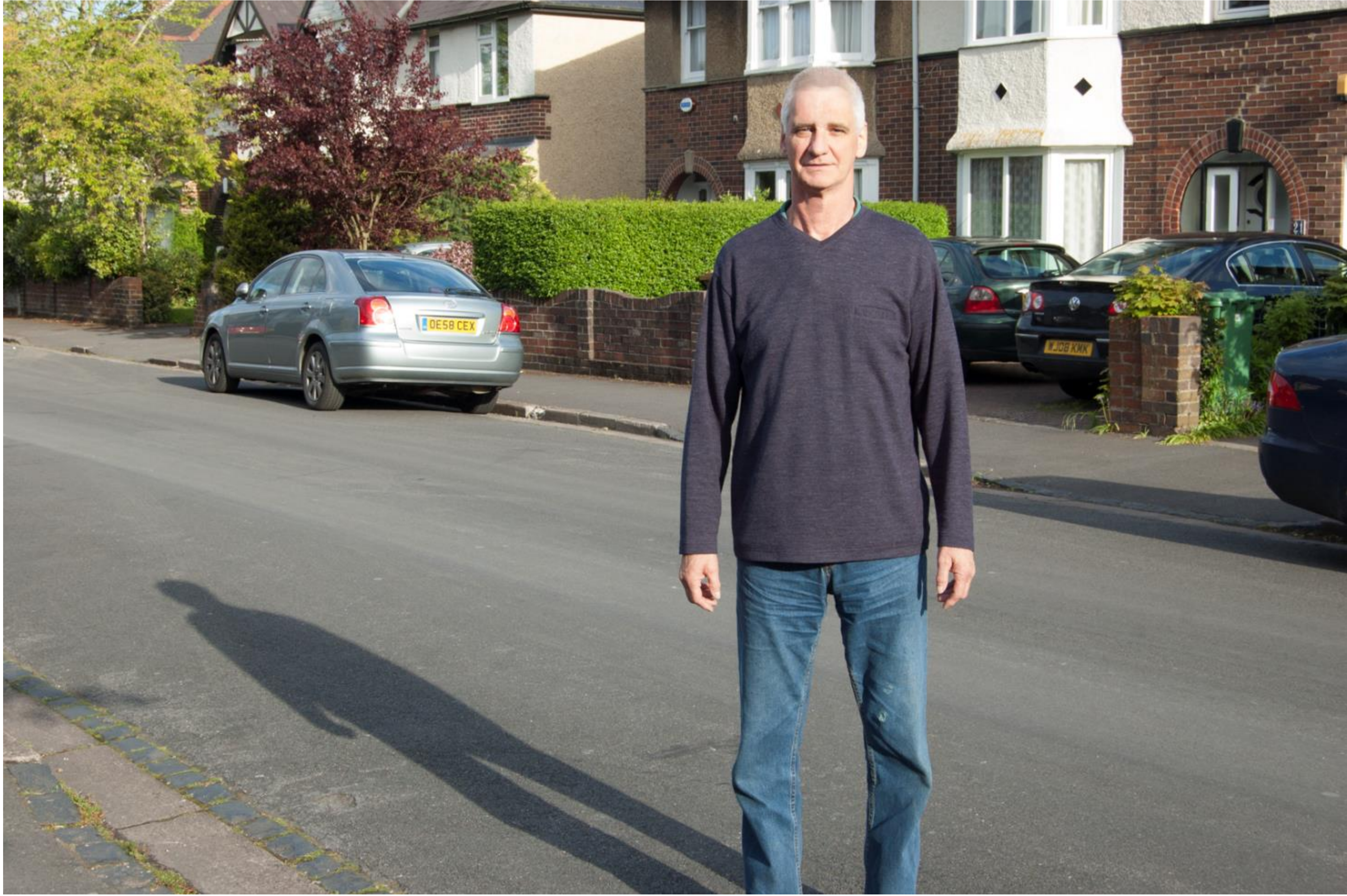

Experiment 2.
Image D.
Addition/
Subtraction—
addition of
rubbish bins

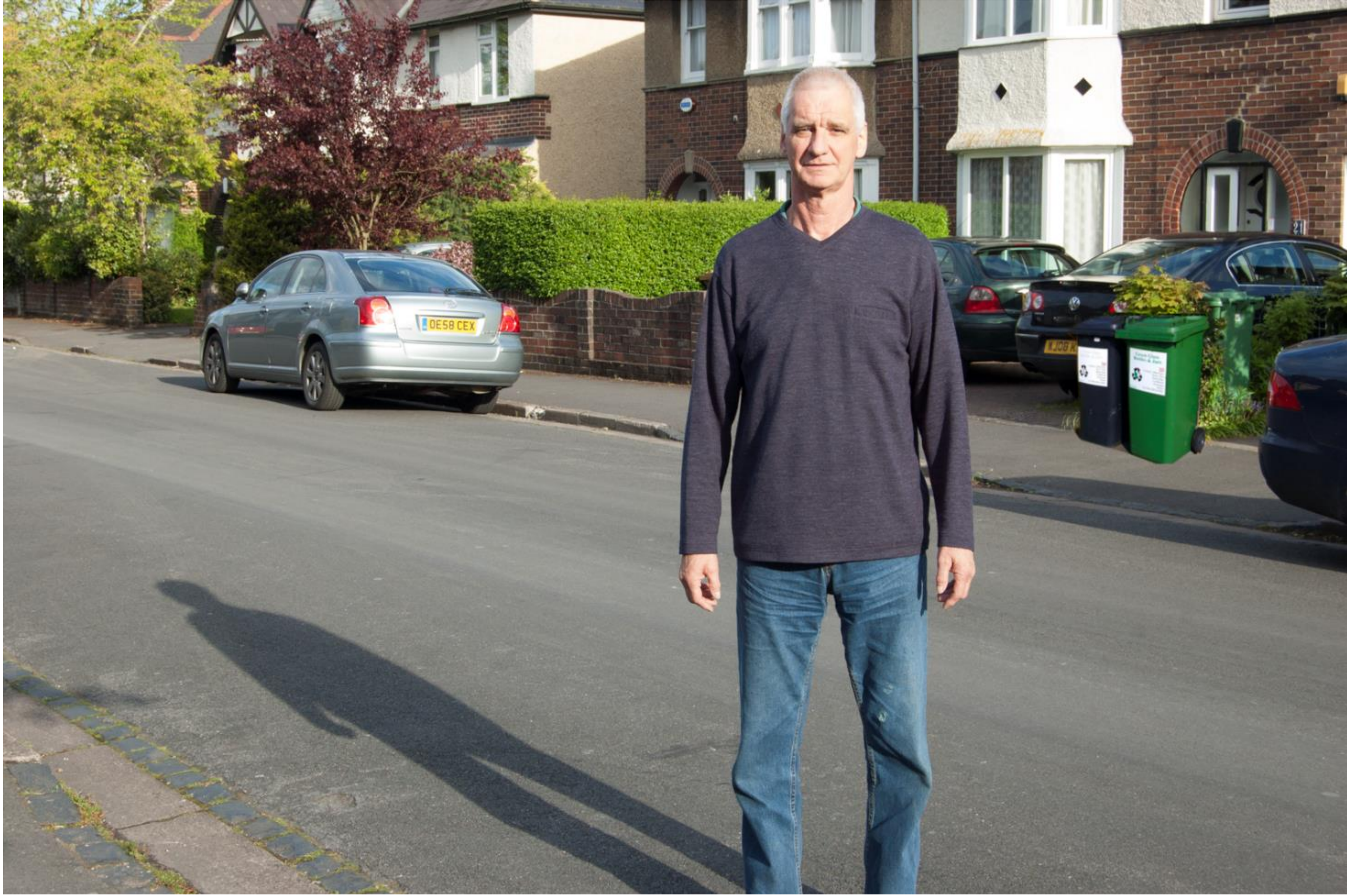

Experiment 2. Image D. Geometry—half of a tree sheered at an angle so that it appears inconsistent with the other half

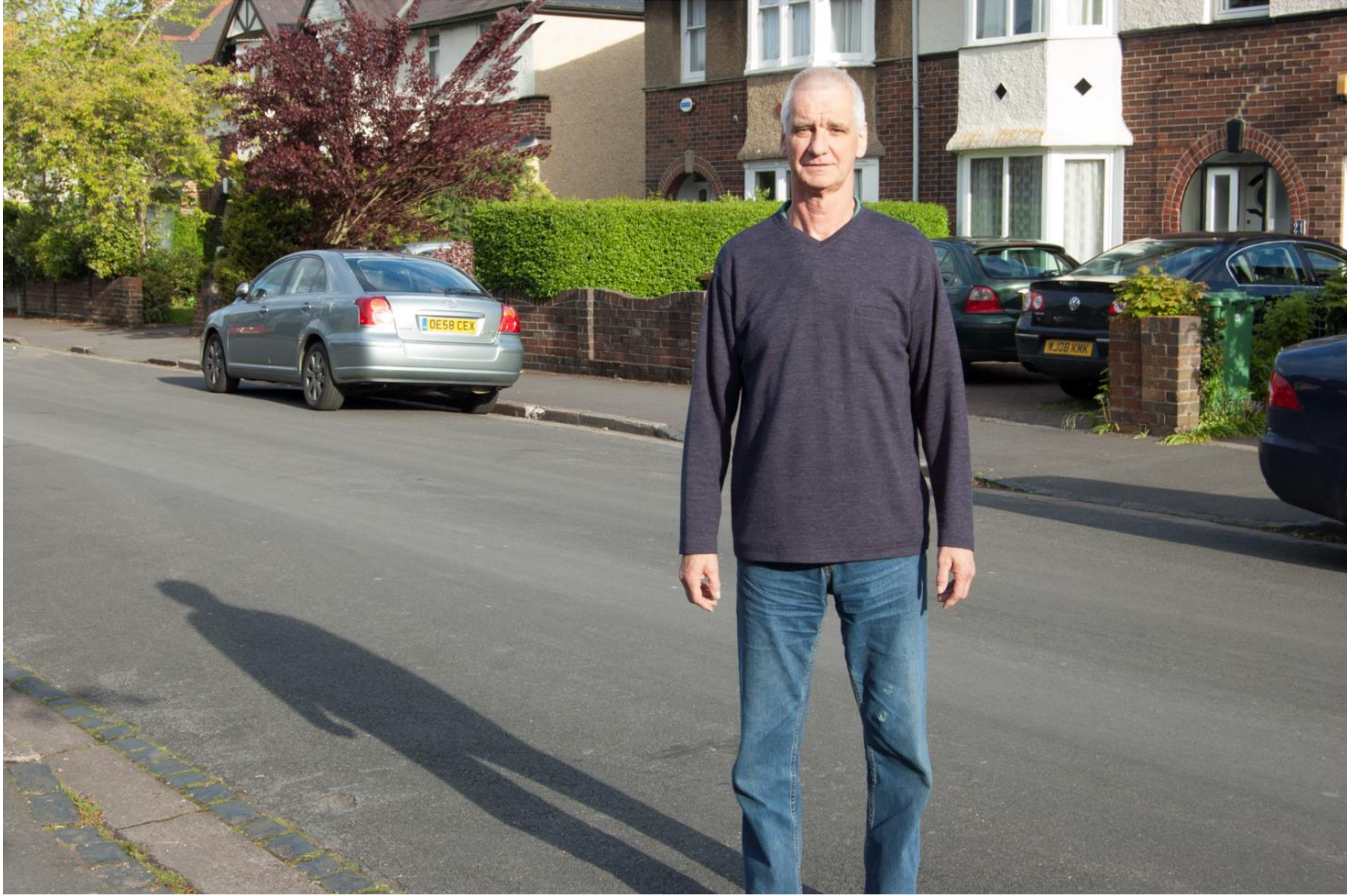

Experiment 2. Image D. Shadow—the man’s shadow has been moved so that it is inconsistent with the lighting for the rest of the scene

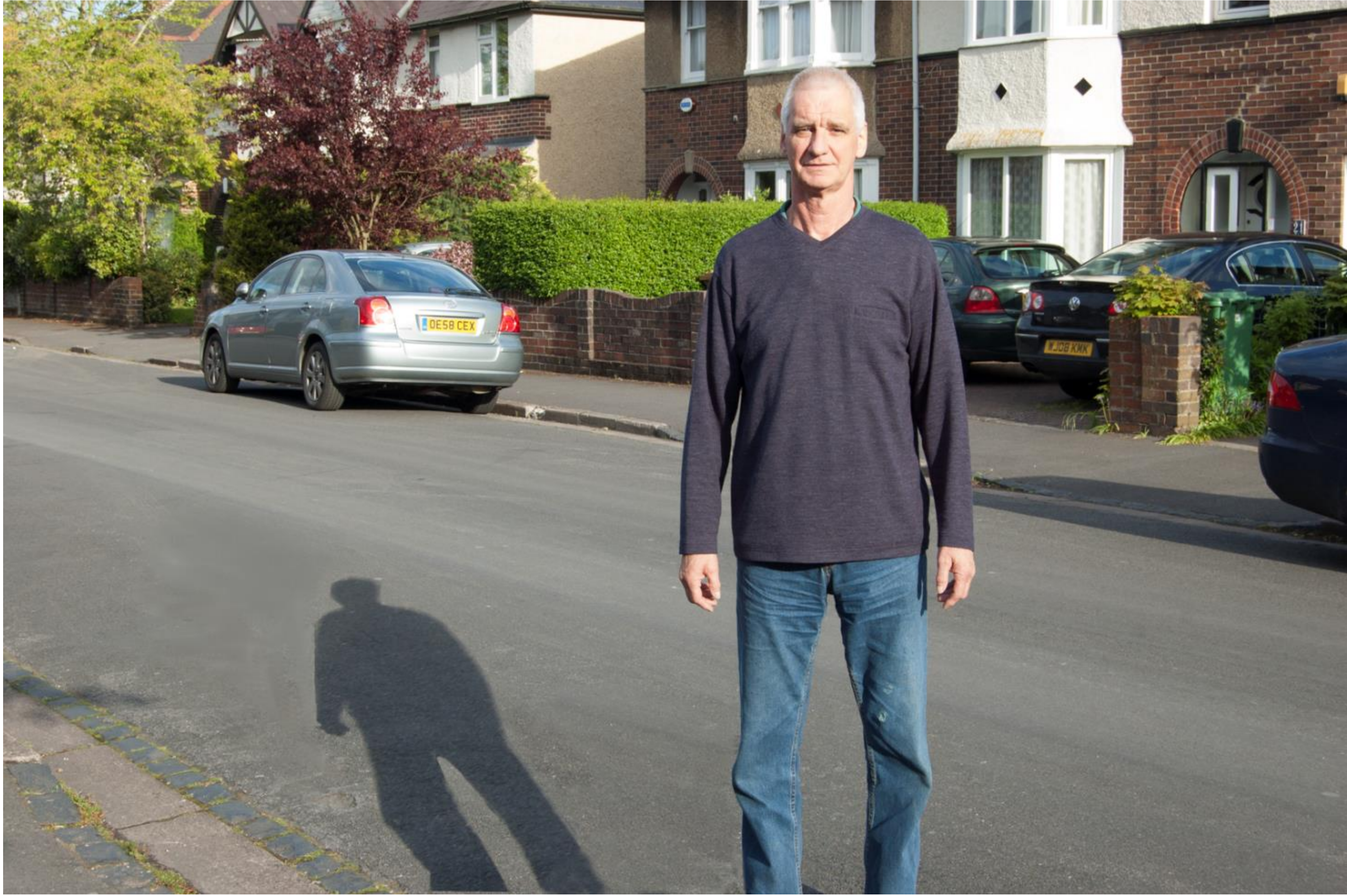

Experiment 2.
Image D.
Super-Additive—combination of all previously described manipulations

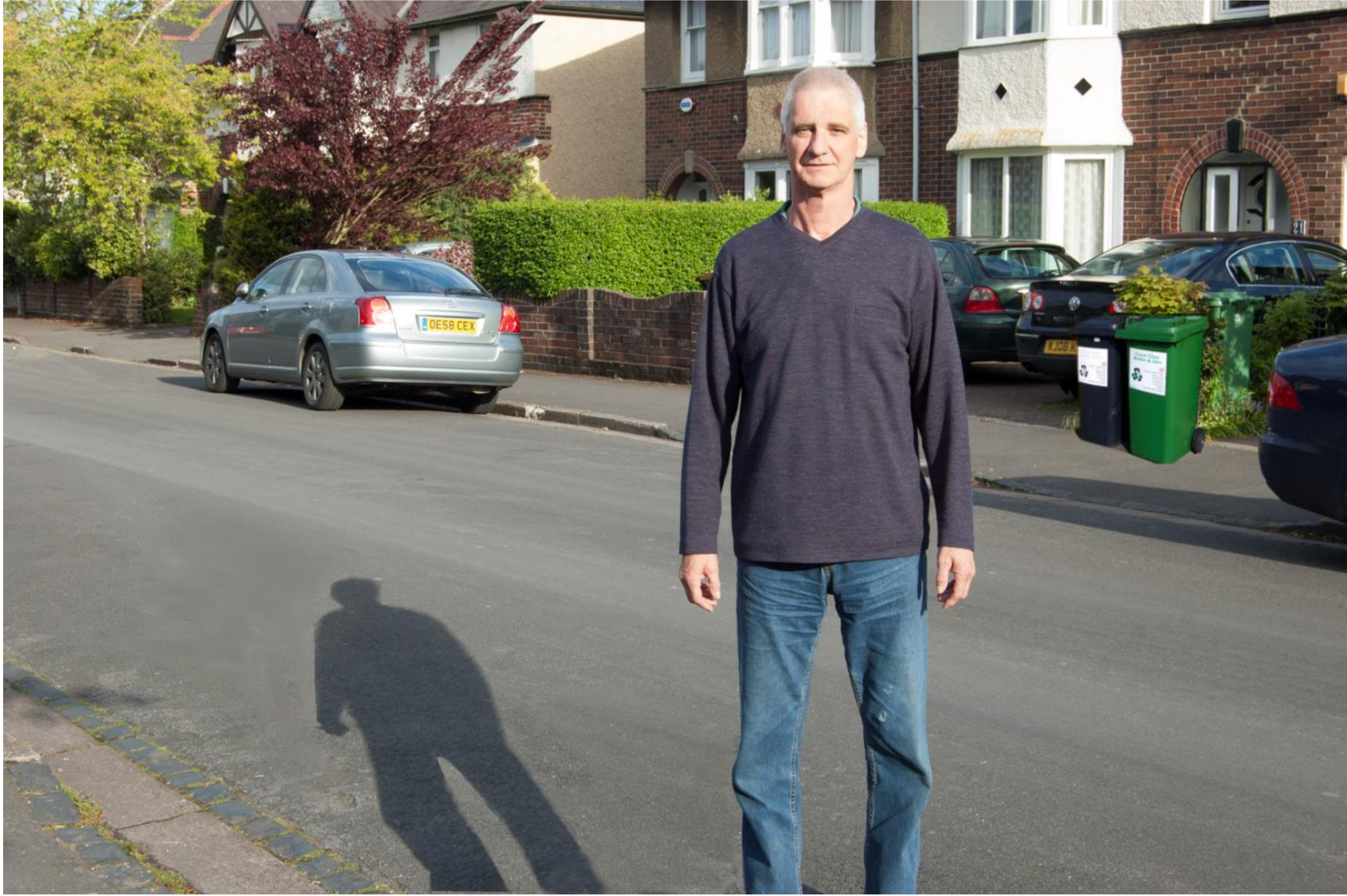

Supplement: Supplementary file 1 — Examples of the images used in Experiments 1 and 2. (PDF 5902 kb) [file 41235_2017_67_MOESM1_ESM.pdf]
