# Supplementary material for: Can people identify original and manipulated photos of real-world scenes?
Source: Cogn Res Princ Implic. 2017 Jul 18;2:30. doi: 10.1186/s41235-017-0067-2 (PMC5514174; doi:10.1186/s41235-017-0067-2)
Supplement: Supplementary file 2 — Details of the saliency analyses for the stimuli used in Experiments 1 and 2. (DOCX 333 kb) [file 41235_2017_67_MOESM2_ESM.docx]

**Additional files**

**Experiment 1**

**Saliency analyses.** The extent to which certain parts of a scene stand out relative to other parts (object or region saliency) can affect the distribution of visual attention. We ran two saliency analyses to check whether our manipulations influenced the salience of the region where the manipulation had been made.

We chose two of the various models available to compute bottom-up predictions of visual salience; the classic Itti-Koch model (Itti & Koch, 2000; Itti, Koch, & Niebur, 1998) and the Graph-Based Visual Saliency (GBVS) model (Harel, Koch, & Perona, 2006). For simplicity, we will refer to these models as IK and GBVS.

***IK.*** Using Matlab, we ran the IK model on the six original images and the 30 manipulated versions of these images. For each image, the model created a saliency map with a saliency value for each individual pixel. Recall that in the location task of Experiment 1, subjects saw the image with a 3×3 grid overlaid and were asked to select the region that they believed had been manipulated. Therefore, to quantify the saliency values, we calculated the mean saliency for each of the nine regions for each the 36 images. We then checked whether our manipulations of the images had affected the saliency of the manipulated region compared with the same region in the original image. That is, did our manipulations make the regions any more or less salient than they were in the original image? Figure S1 shows the mean saliency of the *manipulated* region alongside the mean saliency of that same region in the *original* image. As shown, our manipulations had no systematic effect.


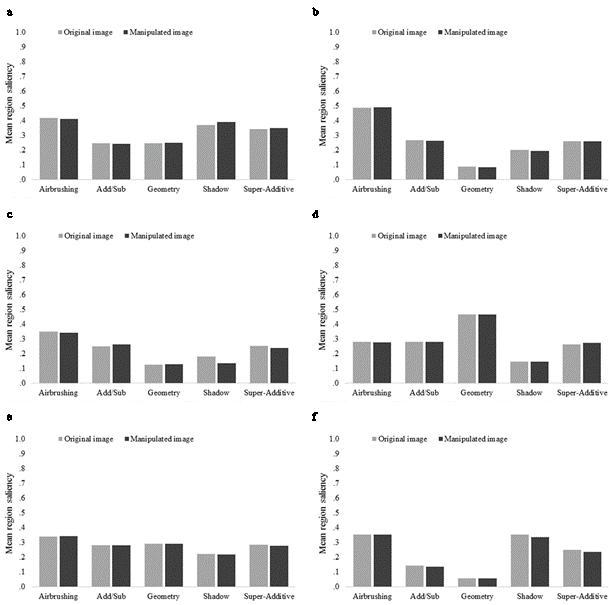


*Figure S1*. Mean saliency of the manipulated region in the manipulated image and mean saliency of the same region but in the original image, where higher values indicate a more salient region. Data is shown for each of the 6 images, a-f, and for all five manipulation types, computed using the IK model.

***GBVS.*** Next, in Matlab we ran the same analysis using the GBVS model to determine whether our manipulations had influenced region salience. Replicating the results from the IK model, Figure S2 shows that our manipulations made little difference to the salience of the image regions.


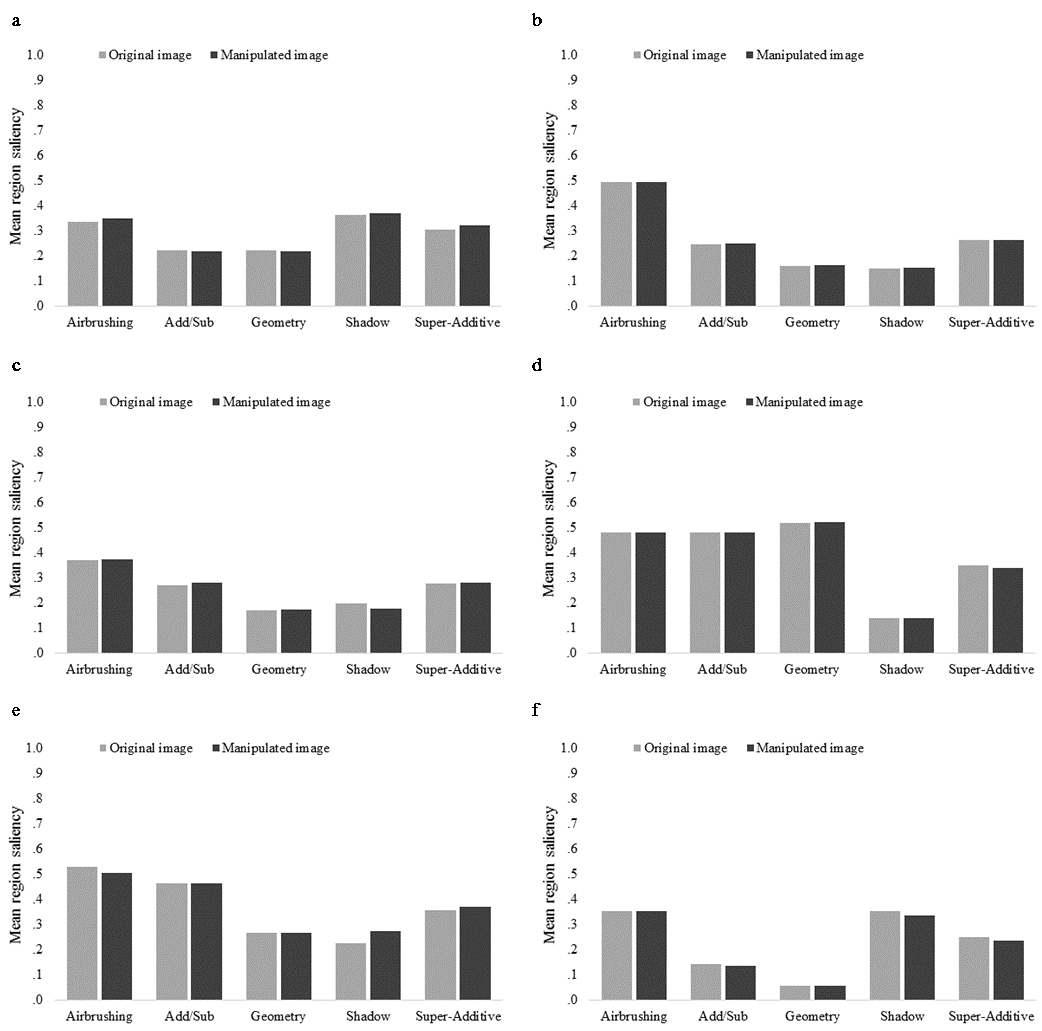


*Figure S2.* Mean saliency of the manipulated region in the manipulated image and mean saliency of the same region but in the original image, where higher values indicate a more salient region. Data is shown for each of the 6 images, a-f, and for all five manipulation types, computed using the GBVS model.

**Experiment 2**

***IK.*** We ran the IK model on the six original images and the 30 manipulated versions of these images. For each image, the model created a saliency map with the saliency value for each individual pixel. Recall that in Experiment 2 the location task used a 4×3 overlaid grid. Therefore, to quantify the saliency values, we calculated the mean saliency for each of the 12 regions. We calculated these mean values for each region in each of the 36 images.

As in Experiment 1, we considered whether our manipulations of the images had made any difference to the saliency of the manipulated region compared with that same region in the original image. Figure S3 shows a similar pattern of results to those observed in Experiment 1—our manipulations had little or no systematic effect on region salience.


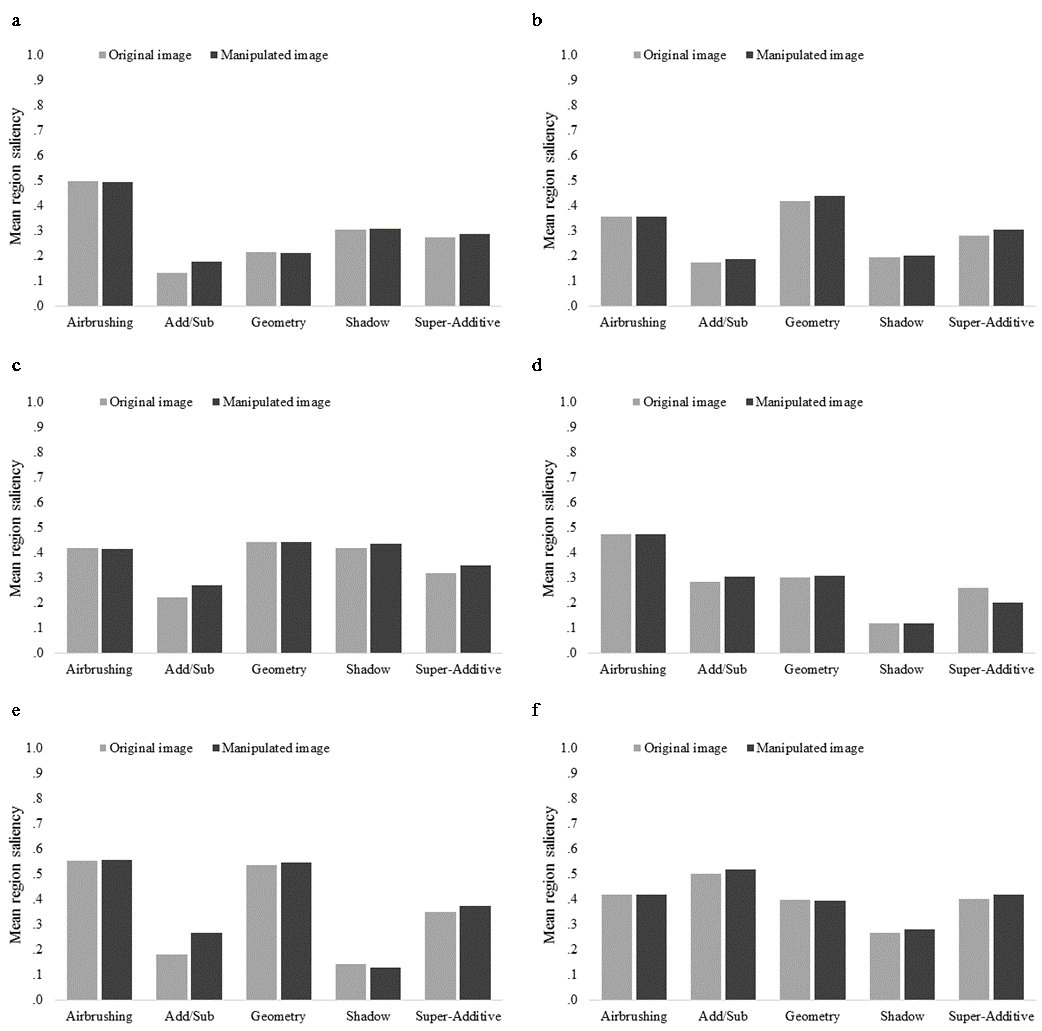


*Figure S3*. Mean saliency of the manipulated region in the manipulated image and mean saliency of the same region but in the original image, where higher values indicate a more salient region. Data is shown for each of the 6 images, a-f, and for all five manipulation types, computed using the IK model.

***GBVS.*** Next we ran the GBVS model on all six original and the 30 manipulated images. Again, we considered whether our manipulations made the regions any more or less salient than they were in the original image. Replicating our previous results, Figure S4 shows that our manipulations made little difference to the salience of the images. In sum, as in Experiment 1, our saliency analyses indicate that we did not influence people’s accuracy on the detection and location tasks by inadvertently changing the salience of the manipulated regions.


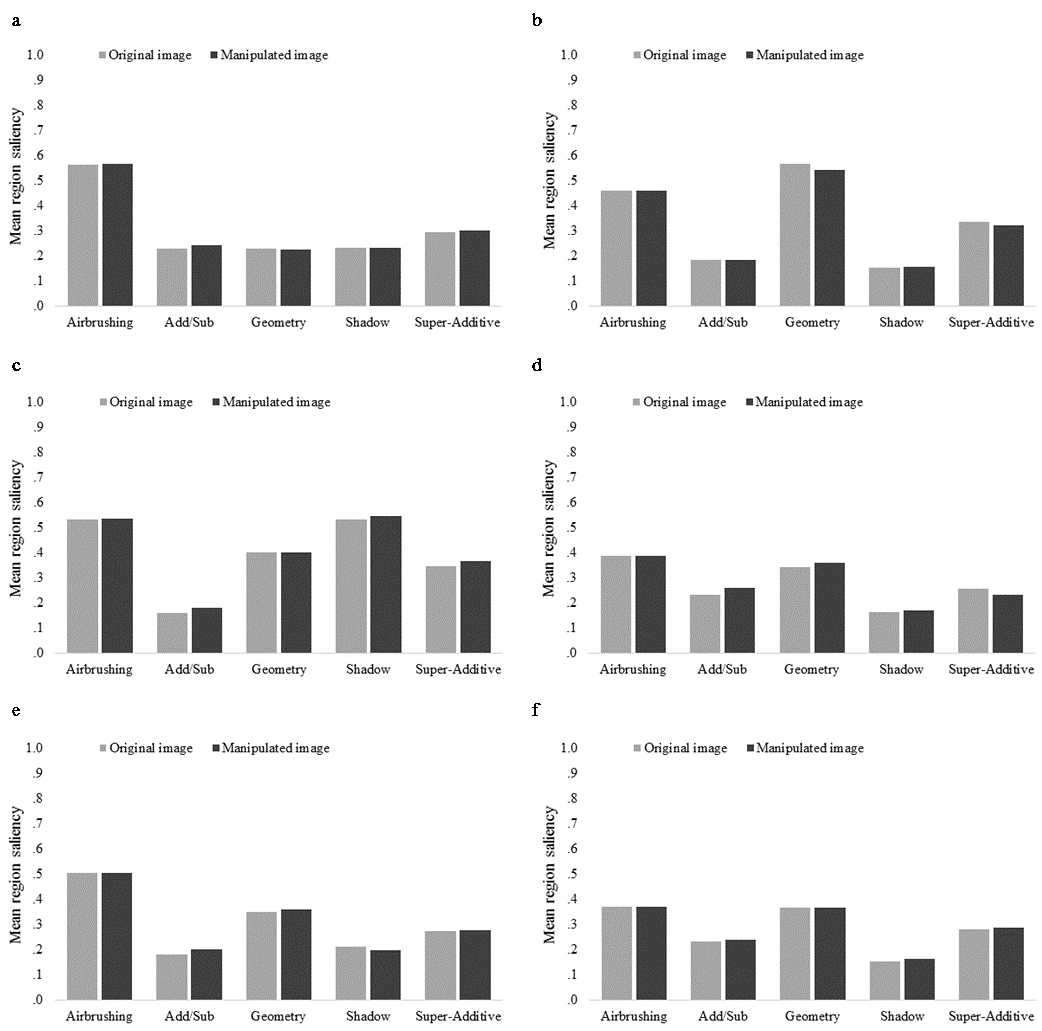


*Figure S4*. Mean saliency of the manipulated region in the manipulated image and mean saliency of the same region but in the original image, where higher values indicate a more salient region. Data is shown for each of the 6 images, a-f, and for all five manipulation types, computed using the GBVS model.
